# Comprehensive Overview of Alzheimer’s Disease: Etiological Insights and Degradation Strategies

**DOI:** 10.3390/ijms25136901

**Published:** 2024-06-24

**Authors:** Manish Kumar Singh, Yoonhwa Shin, Songhyun Ju, Sunhee Han, Sung Soo Kim, Insug Kang

**Affiliations:** 1 Department of Biochemistry and Molecular Biology, School of Medicine, Kyung Hee University, Seoul 02447, Republic of Korea; manishbiochem@gmail.com (M.K.S.); jac03032@khu.ac.kr (Y.S.); thdgus8543@khu.ac.kr (S.J.); sunheehan@khu.ac.kr (S.H.); 2 Biomedical Science Institute, Kyung Hee University, Seoul 02447, Republic of Korea; 3 Department of Biomedical Science, Graduate School, Kyung Hee University, Seoul 02447, Republic of Korea

**Keywords:** Alzheimer’s disease, amyloid-β, autophagy, GWAS, proteases, lysosome, tau

## Abstract

Alzheimer’s disease (AD) is the most prevalent neurodegenerative disorder and affects millions of individuals globally. AD is associated with cognitive decline and memory loss that worsens with aging. A statistical report using U.S. data on AD estimates that approximately 6.9 million individuals suffer from AD, a number projected to surge to 13.8 million by 2060. Thus, there is a critical imperative to pinpoint and address AD and its hallmark tau protein aggregation early to prevent and manage its debilitating effects. Amyloid-β and tau proteins are primarily associated with the formation of plaques and neurofibril tangles in the brain. Current research efforts focus on degrading amyloid-β and tau or inhibiting their synthesis, particularly targeting APP processing and tau hyperphosphorylation, aiming to develop effective clinical interventions. However, navigating this intricate landscape requires ongoing studies and clinical trials to develop treatments that truly make a difference. Genome-wide association studies (GWASs) across various cohorts identified 40 loci and over 300 genes associated with AD. Despite this wealth of genetic data, much remains to be understood about the functions of these genes and their role in the disease process, prompting continued investigation. By delving deeper into these genetic associations, novel targets such as kinases, proteases, cytokines, and degradation pathways, offer new directions for drug discovery and therapeutic intervention in AD. This review delves into the intricate biological pathways disrupted in AD and identifies how genetic variations within these pathways could serve as potential targets for drug discovery and treatment strategies. Through a comprehensive understanding of the molecular underpinnings of AD, researchers aim to pave the way for more effective therapies that can alleviate the burden of this devastating disease.

## 1. Introduction

Alzheimer’s disease (AD) stands as the most prevalent form of dementia among the elderly, with other types including frontotemporal dementia, Lewy body dementia, and vascular dementia [1,2,3,4]. As AD progresses, it primarily impacts brain regions like the hippocampus and prefrontal cortex and disrupts cognitive functions [1]. Studies have reported the extracellular accumulation of amyloid-β (Aβ)1–42 peptides and intracellular neurofibrillary tangles (NFTs) comprising Aβ oligomers and phosphorylated tau proteins. The disease progression worsens brain functions and leads to synaptic loss and memory impairment [5]. In healthy individuals, tau proteins are typically phosphorylated at around 10 sites [5,6,7]. However, in AD patients, tau phosphorylation extends to 40–45 sites [6]. Consequently, hyperphosphorylated tau loses its ability to bind to microtubules and is thus transported to different parts of neurons, such as soma, and dendrites spines, disrupting the synaptic connections among them. Molecular and genetic studies in AD patients have identified three primary genetic mutations associated with Early-Onset Alzheimer’s Disease (EOAD), linked with genes encoding amyloid precursor protein (APP), presenilin 1 (PSEN1), and presenilin 2 (PSEN2) [8]. However, the APOEε4 allele is considered the predominant genetic risk factor for AD, notably in sporadic AD cases referred to as Late-Onset Alzheimer’s Disease (LOAD). Additionally, variations in CD33 splicing play a significant role in altering and influencing this genetic predisposition [9,10]. Several tests used for diagnosing AD clinically include brain imaging techniques such as positron emission tomography (PET), cerebrospinal fluid (CSF)-based biomarkers like Aβ1–42, p-Tau, and neurogranin-a synaptic protein, as well as blood test-based biomarkers such as proinflammatory cytokines including IL-6, ICAM-1, and VCAM-1 [11]. Furthermore, genome-wide association studies (GWASs) conducted across diverse populations have implicated rare non-synonymous variants like TREM2, PLCG2, and ABI3 in LOAD [12]. Additionally, inflammatory pathways and various neuroinflammation-modulated signaling pathways play significant roles in exacerbating Aβ toxicity and AD pathology. Thus, understanding these genetic and molecular intricacies is vital for developing targeted therapies aimed at mitigating the devastating effects of AD on cognitive function and quality of life [13].

Existing evidence has revealed that various enzymes are dysregulated in AD, which is involved in the cleavage of amyloid precursor protein (APP) and tau hyperphosphorylation, resulting in increased levels of amyloid-β and tau fibrils in neurons and, ultimately, neurodegeneration. This process includes numerous endosomal–lysosomal proteases, such as cathepsins, calpains, caspases, and matrix metalloproteinases (MMPs), which exhibit a crucial role in tau cleavage and contribute to AD pathology [14]. Consequently, several small molecule inhibitors targeting these proteases and kinases are currently under clinical trials for treatment, aiming to inhibit the aggregation of amyloid-β and tau fibrils in AD patients. Here, we comprehensively elucidate the important genetic factors and degradation pathways implicated in the prevention of AD. Our object is to shed light on the genetic risk factors, both high and low, and signaling pathways involved in Aβ cytotoxicity. Additionally, we examine key degradation pathways, including the Receptor for Advance Glycation End Product (RAGE), Endoplasmic Reticulum-Associated Degradation (ERAD), and autophagy, which play crucial roles in the clearance of toxic Aβ plaques and NFTs from the brain. We aim to highlight the genetic risk factors and neuronal markers that warrant further investigation as potential early biomarkers and therapeutic targets that can be utilized in the development of therapeutics and strategies to manage the progression of AD effectively.

## 2. Methods

This review outlines potential strategies based on GWAS data in AD. We reviewed an enormous amount of the literature on GWASs derived from transcriptomic, expression QTL (eQTL), methylation QTL (mQTL), and methylome data. Identifying potential candidate SNPs as risk factors for disease etiology was challenging because the significance threshold (*p*-value < 0.05) was difficult to meet. Understanding the causal genetic variants and genes influencing AD risk on susceptible loci remains limited, with only a few genes extensively studied in AD patients. Despite a GWAS with a large population size identifying a locus covering 3% of AD cases, attributing heritability to these loci is still ambiguous.

It was difficult to provide detailed information about each genetic locus, the affected genes, and subsequent proteins associated with AD. This review encompassed data from the last two and a half decades, sourced from the AD forum and the World Health Organization (WHO). These data were collected through extensive searches of online databases from January 2021 onwards such as https://www.alzforum.org, https://forum.alzheimers.org.uk, and https://www.who.int/data for gathering literature. For research articles, Medline https://www.nlm.nih.gov/medline, https://clarivate.com/webofscience-medline, PubMed https://pubmed.ncbi.nlm.nih.gov, and Google Scholar https://scholar.google.com databases were used to find the most relevant and recent publications. The most important and recent research articles from each relevant author in AD and tauopathy were included. The collected research allowed the inclusion of detailed information related to GWASs across various populations, focusing on the most relevant genes and data related to mechanisms affecting Aβ production and clearance of Aβ. Data not directly linked with AD but rather other forms of dementia were excluded. This review primarily focuses on the disease etiology based on GWASs and degradation pathways for early biomarkers and therapeutic perspectives. A detailed discussion of each section with significant mechanisms is provided, and references are cited for all encoded statements in this review.

## 3. Alzheimer’s Disease Etiology

### 3.1. Amyloid-β Production, Tau Hyperphosphorylation, and Molecular Biomarkers

**Figure 1:**
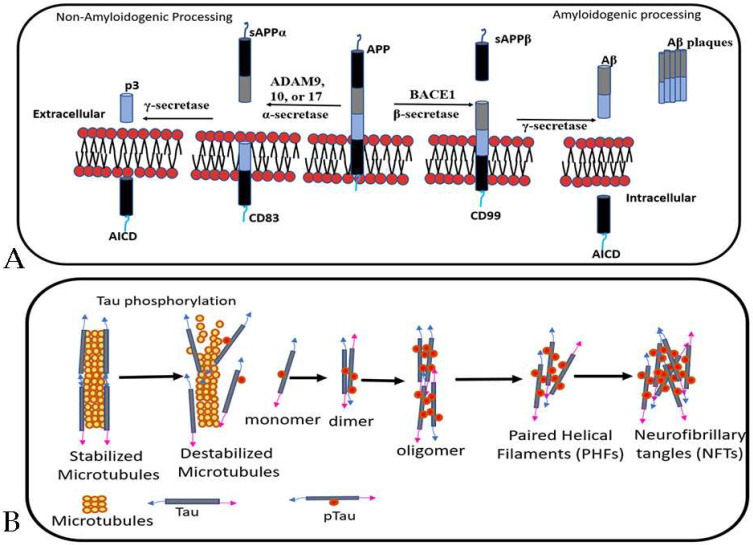
This image illustrates the processing of amyloid precursor protein (APP) and the subsequent formation of amyloid plaques (**A**) as well as tau hyperphosphorylation and the formation of neurofibrillary tangles (**B**). (**A**). Processing of APP involves two pathways including the non-amyloidogenic and amyloidogenic pathways. In the non-amyloidogenic pathway, α-secretase cleaves APP, while in the amyloidogenic pathway, β-secretase and γ-secretase are involved in the cleavage process, leading to the release of Aβ into the extracellular space. The initial cleavage of APP by proteases releases the APP intracellular domain (AICD) into the intracellular space. (**B**). The tau protein normally binds to microtubules, stabilizing their structure. However, hyperphosphorylation of tau leads to the release of tau filaments and destabilization of the microtubule structure. This results in the formation of various tau aggregates, including dimers, oligomers, paired helical filaments (PHFs), and neurofibrillary tangles (NFTs). Adapted from Ref [17].

Amyloid-β (Aβ) is a peptide fragment of amyloid precursor protein (APP) that undergoes enzymatic processing by two enzymes, β-secretase and γ-secretase. β-secretase is a transmembrane aspartate protease, also termed β-site APP-cleaving enzyme-1 (BACE1) [15]. Similarly, γ-secretase is also an aspartate protease composed of four subunits including presenilin (PSEN), Nicastrin (NCSTN), anterior pharynx defective 1 (APH1), and presenilin enhancer-2 gene (PSEN-2) [16]. The cleavage of APP by β-secretase (BACE1) generates a membrane fragment called C99 and, followed by α-secretase action, generates shorter soluble Aβ1–15 and Aβ1–16 peptides at the ectodomain (Figure 1) [17]. Conversely, cleavage at the C-terminal intramembranous site by γ-secretase generates insoluble Aβ peptides, leading to the formation of Aβ plaques in the extracellular space and cytoplasm. Several studies on the inhibition of these proteases have implicated reduced Aβ peptides in vivo AD models. In LOAD, the levels of Aβ1–42 and tau hyperphosphorylation are increased significantly. Tau, a microtubule-associated protein tau (MAPT), stabilizes neuronal structure and integrity by binding to tubulin molecules [18]. Several kinases such as glucose synthase kinase-3 (GSK-3), cyclin-dependent protein kinase-5 (cdk5), protein kinase A (PKA), calcium calmodulin-dependent protein kinase-II (caMKII), tau tubulin kinase, a dual serine/threonine, and tyrosine kinase, expressed in human brains and multiple tissues such as the spinal cord and testis. These kinases modulate the phosphorylation status of tau in LOAD patients [19,20].

Mutational studies using GWAS data analysis identified APOE-ε4 as a predominant risk factor, which is strongly associated with typical late-onset AD, albeit with low penetrance. APOE exhibits three isoforms, i.e., ε2, ε3, and ε4, wherein APOEε4 increases AD risk in a dose-dependent manner. Conversely, ApoE-ε2 provides protection against the disease risk conferred by the APOEε4 allele [21]. APOE2 is the least common allele variant in AD and is linked to elevated levels of tau and phosphorylated tau in APOE2-TR mice overexpressing human Tau^P301L^-APOE2. Single Recognition Particle 14 (SRP14), a ribonucleoprotein complex targeting secretory proteins to the endoplasmic reticulum is upregulated in APOE2 carriers compared with non-carriers, highlighting its critical role in tau pathology and Aβ deposition in AD in vivo [22]. Rare variants in APOE3 with mutations in the lipid-binding or lipoprotein receptor-binding regions decrease the risk of AD to a comparable extent as APOE2 [1]. Notably, the APOE-ε3 V236E Jacksonville variant (APOE3-Jac) reduces fibrillar Aβ plaques, whereas the APOE3 R136S Christchurch (APOE3ch) reduces cognitive decline in patients with an autosomal AD mutation [23]. Conversely, APOE2 carriers exhibit delayed Aβ deposition, later clinical onset, and increased longevity compared with non-APOE2 carriers [22]. Genetic evidence indicates that individuals carrying the APOE-ε4 allele exhibit higher Aβ deposition compared with APOE ε3/ε4 allele carriers [24]. Mutations in the PSEN1 and APOE genes can lead to the increased formation of Aβ1–42 and the production of aggregated oligomers [25]. APOE gene mutations, particularly APOEε4 and APOE2, play roles in Aβ metabolism and lipid transport within the brain [26].

The APOE-ε4 gene significantly affects the splicing of CD33, while APOE2 gene variants are associated with the regulation of HMOX-1 expression and possess antioxidant properties relevant to AD [27]. APOE2 carriers show increased levels of SLAM family member 8 (SLAMF8), a CD2 family member, implicated in modulating reactive oxygen species and inflammation in the brain, as well as influencing macrophage function and supporting the growth of neoplastic mast cells via SHP-2 [28]. Recent findings have demonstrated that APOEε4 loss-of-function is associated with a high risk of AD. However genetic analysis has revealed that ε4 drives AD risk via a gain in abnormal function rather than a loss of function in AD pathogenesis. Supporting this hypothesis, studies have found that APOEε4 increases tauopathy and neurodegeneration by promoting lipid accumulation and impairing cholesterol metabolism in a tauopathy model [24,29]. The precise role of APOE in AD pathogenesis remains elusive, and further studies are warranted to investigate this potential mechanism in large populations carrying APOE mutations.

Tauopathies manifest as progressive cognitive and motor impairment, encompassing conditions such as progressive supranuclear palsy, corticobasal degeneration, chronic traumatic encephalopathy, and certain forms of frontotemporal dementia. Genetic evidence from model organisms like mice suggests that tau plays a pivotal role in age-related neurodegeneration. However, fewer studies have demonstrated tau protein’s involvement in progressive synaptic dysfunction and neuronal loss, likely stemming from various cellular derangements including oxidative and immune-mediated injury, altered proteostasis, aberrant transcription, and post-translational modifications [18]. Tau, primarily localized in the axons of healthy neurons, relocates to the soma and dendrites under pathological conditions [30]. Axons play a crucial role in maintaining neuronal function by facilitating anterograde and retrograde transport of cargo [31]. The mechanisms underlying tau seeding and spreading remain elusive, but some in vivo studies suggest the involvement of either exocytosis through vesicles or inflammation. The relevance of these mechanisms in AD pathogenesis remains unclear. Studies in transgenic mice have linked the tau-mediated reduction of kinesin-1 light chain to impaired anterograde axonal trafficking [32]. In AD, hyperphosphorylation of tau increases, leading to the formation of neurofibrillary tangles (NFTs) [11]. Tau-induced neuronal damage initiates in the entorhinal cortex and progressively spreads to the hippocampus and other cortical regions. Later, tau oligomers spread to other areas of the brain such as the corpus callosum and the mediolateral axis [33,34]. Additionally, extracellular oligomeric tau has been implicated in memory impairment and cognitive dysfunction in mice [35]. Mutations in MAPT genes are reported in familial frontotemporal dementia (FTD), characterized by prominent neurofibrillary tangle deposition [18].

Several studies have highlighted the cerebrospinal fluid (CSF) tau level as an early indicator of Aβ pathogenesis. However, plasma Aβ also changes in AD patients compared with controls, and the difference between amyloid-positive and -negative individuals is a relatively small-fold change [36]. Recent research, utilizing positron emission tomography (PET) analysis on CSF samples from Alzheimer’s disease (AD) patients, identified three distinct phosphorylated tau (p-tau) epitopes, including p-tau 181, p-tau 217, and p-tau 231, evident across clinical, preclinical, and pre-amyloid phases of AD [37,38]. Among these, p-tau 231 exhibited a significant increase, accumulating notably in brain regions associated with the default mode network, such as the precuneus, posterior cingulate cortex, and orbitofrontal cortex [39]. As a result, cerebrospinal fluid (CSF) and brain regions affected early in AD progression, such as the cortex and hippocampus, indicate that CSF p-tau levels and Aβ PET are pivotal markers for the early detection of AD [40]. A parallel study revealed elevated p-tau 217 and p-tau 181 levels in aggregated Aβ, detectable preceding the onset of aggregated tau pathology [41]. This finding was supported by another study where p-tau 181 was elevated in the CSF of patients with amyloid pathology and Braak NFT ≥ III detected by Aβ and Tau PET screening in early AD stages, confirming an escalating trend with disease progression [42,43]. Notably, 18F-fluorodeoxyglucose (FDG) PET has been employed to differentiate between typical and atypical AD based on distinctive frontal and parietal atrophy and hypometabolism [44]. Moreover, the levels of p-tau forms such as p-tau 205 and total tau surged with the emergence of LOAD symptoms [45]. Notably, Phan and Cho developed aptamer-mediated biosensors tailored to specifically detect p-tau at threonine 231, a crucial early p-tau isotope frequently utilized in the diagnosis of AD [46]. Additionally, Gonzalez-Ortiz et al. demonstrated that plasma brain-derived tau (BD-Tau) with p-tau alongside Aβ42/Aβ40 as a blood-based based-biomarker for diagnosis of AD. This approach enhances agreement with autopsy results or those derived from CSF or neuroimaging biomarkers [47,48]. Other blood biomarkers for AD including GFAP and β-synuclein are significantly observed in AD [49,50]. Various studies have demonstrated that combined biomarkers provide better diagnostic accuracy for AD than individual measures alone [51]. For instance, the combination of three plasma biomarkers such as APP669–711 with Aβ and p-tau217, plasma Aβ42/Aβ40, and plasma NFL showed improved diagnostic performance when the APOE genotype was included [52,53,54]. Further studies that analyze combined biomarkers, including plasma Aβ42/Aβ40 and other measurements, may confer even more accurate diagnoses from blood samples, representing a valuable avenue for future investigation.

Additionally, modifiers of p-tau levels, such as the α7 subtype of nicotinic acetylcholine receptors (α7nAChRs), were found to induce tau phosphorylation at specific sites (Ser 199, Ser 396, and Thr 205) in the hippocampus of 12-month-old α7 knockout mice. Interestingly, these mice exhibited increased levels of APP and Aβ without the formation of senile plaques, suggesting a potential role for α7nAChRs within the tripartite interplay involving α7nAChRs, Aβ, and tau [55]. Increased oxidative stress leads to elevated level of GSK-3β and protein kinase, and phosphatase PP2A activates tau phosphorylation at ser396, ser404, and thr231. In the AD hippocampus, the decreased expression of peptidyl prolyl cis-transferase (PIN1) and increased p-tau level act as an additive factor in AD pathogenesis [56]. This observation is reinforced by subsequent experiments using cortical neuronal cells with H_2_O_2_, which results in the activation of GSK-3β and PIN1. Thus, kinase inhibitors could be beneficial in ameliorating p-tau in AD. Some kinase inhibitors including Tideglusib and lithium, are utilized to modulate GSK-3β activity, although clinical trials are ongoing in AD and other types of dementia [57]. Therefore, further investigations are warranted to gain deeper insights into the pathological and non-pathological conditions of p-tau and NFT production in AD and related tauopathies.

### 3.2. GWAS and Genetic Risk Factors Associated with AD and Synaptic Plasticity

High-throughput genomic analysis such as genome-wide association studies (GWASs) identified several genes linked with disease progression and implicated in synaptic dysfunction and memory impairment [58]. GWAS screening primarily aims to identify susceptible genes and investigate their impact on AD pathology and early detection. These studies provide a novel approach to identifying genetic variations associated with AD, with a significance threshold (*p*-value ≤ 0.05) for single nucleotide polymorphisms (SNPs), indicating a potential risk factor for AD [59]. GWASs are implicated in early biomarkers for various diseases, including cancers and age-related disorders [60,61]. A recent study utilizing the U.K. Biobank for AD familial data, known as GWAS-by-proxy, uncovered 12 genomic loci associated with LOAD [12]. However, our understanding of the causal genetic variants and genes influencing AD risk at these loci remains limited, with only a few genes extensively studied in AD patients.

Despite a large population size exceeding more than 1.1 million individuals and the identification of 38 independent genome-wide significant loci, only 3% of AD cases can be attributed to heritability [62,63]. Notably, novel loci such as rs5011804 at 12p12.1 have shown significant associations with the levels of CDRSB, FAQ, FS fusiform, and ADAS13 in various AD cognitive and neuroimaging analyses, underscoring the necessity for multiple measurements such as neuroimaging data (MRI), fluid biomarkers (blood and CSF), cognitive impairment/dementia, and familial data to establish SNP association as an AD risk factor [64,65]. New AD risk loci have been identified through higher-density genotype imputation, shedding light on candidate causal variants at both new and established risk loci. A GWAS meta-analysis also revealed early-age risk factors in AD brain and lymph node samples such as SORL1, PTK2B, SLC24A4, and ZCWPW1 associated with AD. It is necessary to conduct thorough sequencing studies and in-depth post-GWAS analyses to fully understand the candidate genes and functional variants associated with AD susceptibility. These investigations are crucial in order to determine the functional significance of the identified loci in the pathophysiology of AD [12,66,67]. Furthermore, a robust association was observed between the NYAP1 SNP and PILRA/PILRB protein in the brain, with implications for regulating acute inflammatory reactions in the AD brain [28,68].

Numerous other candidate genes identified in GWASs as AD risk factors are localized on different loci, such as rs6705798 encoding EPS15-homology domain-containing protein1/2 (EHBP1), implicated in Glut4 transportation and expressed in various tissues including the brain. Another SNP, rs73045836, encoding secreted extracellular calcium-binding protein 2 (SMOC2) and its isoform SMOC1, has been identified as a novel biomarker for AD in CSF and brain tissues [26]. In addition to APOE4, other genes associated with AD risk, such as CLU, PICALM, CD33, MS4A4, MS4A6A, TREM2, ABCA7, CD2AP, and EPHA1, have been identified in GWASs using meta-analysis methods involving both AD patients and non-AD populations (Table 1). Among these AD risk genes, the myeloid cell surface antigen CD33, rs3865444, and rs3836656 are linked to microglia-mediated clearance, potentially promoting the accumulation of senile plaques [69,70]. Clusterin (CLU), also known as apolipoprotein J (ApoJ), is a significant risk gene associated with AD that resides in the central nervous system. It is synthesized and released by both astrocytes and neurons in the brain, where it plays a role in regulating lipid metabolism. In the Caucasian population, CLU variants, such as rs93331888 and rs11136000, have been identified [71]. However, in the Asian population no significant associations have been observed, indicating a notable variation in the impact of CLU on AD. Recently, additional candidate loci including BIN1, TREM2, SORL1, MS4A, SPI1, and TOMM40 have also been identified via GWAS meta-analysis (Table 1). These genes demand continued attention and further exploration in the forthcoming studies [70].

Moreover, non-synonymous gene variants are significantly associated with trait associations, with most human trait-associated variants affecting gene expression rather than altering protein-coding sequences. These variants likely mediate their effects via altered gene expression, which may vary depending on cell type [2]. Functional mapping of variants to genes, utilizing positional data and expression quantitative trait loci (eQTL) information from brain and immune tissues/cells, unveiled 989 genes linked to 38 genomic risk loci. These genes predominantly pertain to inflammatory signaling and are associated with immune cells such as microglia, astrocytes, and oligodendrocytes in LOAD patients [60,63]. GWASs identify intergenic regions, wherein all protein-coding genes within 500 kilobases (kb) of the sentinel variant linkage disequilibrium (LD) region (r^2^ > 0.5) are implicated as potential AD risk factors.

Transcriptomic analyses and whole-genome sequencing of brain samples from AD patients have revealed alterations in the expression of genes involved in various signaling pathways. Several mutations and loci associated with AD have been identified through GWASs and collaborations such as the International Genomics of Alzheimer’s Project (IGAP). Over the past two decades, at least 38 loci and more than 300 gene mutations have been linked to AD. Some of the frequently mutated genes in AD cases include CD2AP, APOEε4, PTK2B, CASS4, EPHA1, Zyxin, PACSIN, CD33, and CYP3A (Table 1) [60]. Recent analyses combining eQTL and expression transcriptome-wide association studies (eTWASs) have revealed that the downregulation of EGFR is significantly associated with reduced risk of AD [72]. Conversely, colocalization of eQTL-GWAS and methylation QTL (mQTL) signals has identified TSPAN14, derived from lymphoblastoid cell lines, as being correlated with AD risk [72]. Additionally, pathway enrichment analysis using STRING has identified immune and tumor necrosis factor (TNF)-mediated signaling pathways, involving genes such as SHARPIN, RBCK1, and LUBAC, regulated by OTULIN, as having strong associations with AD risk [72,73].

A study on the Mexican American population with mild cognitive impairment (MCI) revealed various methylation regions between control and MCI patients along with genes implicated in neuronal death, metabolic dysfunction, and inflammatory processes [74]. Numerous environmental factors are associated with epigenetic modification and can be inherited. For instance, exposure to organophosphate pesticides has been shown to promote tau hyperphosphorylation and microtubule dysfunction [75,76]. The methylation of genes, such as ABCA7, BIN1, SORL1, and SLC24A4, is significantly associated with the pathological processing of the tau protein and Aβ peptide in the dorsolateral prefrontal cortex. Additionally, histone acetyltransferase and histone deacetylase inhibitors elevate histone acetylation, thereby exerting various positive effects on AD including preventing memory impairment, cognitive dysfunction, less deposition of the Aβ peptide, and reduced tau phosphorylation and formation of NFTs [77]. Therefore, identifying polymorphisms associated with multiple environmental factors through GWASs could facilitate the development of effective diagnostic and therapeutic strategies.

Furthermore, GWAS data serve as a valuable tool for understanding how AD progression is connected to the development of other diseases. Post-GWAS functional genomic analysis is required to prioritize genes that modulate disease susceptibility and identify candidate causal genes for further functional validation in AD animal models [78]. For instance, Lee and colleagues used blood samples from AD patient and found associations between the expression of GPBP1, also known as *Vasculin*, in both vascular wall and plasma, crucial for atherosclerosis, and SETDB2, a SET-domain-containing lysin methyltransferases involved in lipid metabolism via the glucocorticoid-dependent pathway. These genes have disease-related signatures both in AD and cardiovascular disease (CVD), facilitating the implementation of personalized prevention strategies [79]. Further analysis showed that the expression of GPBP1 and SETDB2 is correlated with the tau level in AD mice [80]. Bellenguez et al. conducted a comprehensive search for potential causal genes across 40 novel loci and found that 51% of the AD loci contain candidate causal genes related to myeloid cell function [72]. Although these genes are involved in various biological processes, the exact mechanism through which they contribute to the pathogenesis of AD remains elusive.

**Table 1:** Genetic risk factors associated with AD and synaptic dysfunctions.

Genes	Localization	Functions	References
Apolipoprotein E (APOE)	Three human APOE isoforms—ε2, ε3, and ε4—secreted from microglia, astrocytes, and other neurons	Binds to Aβ to facilitate its uptake and clearance by microglia	[21,29,81,82,83,84]
A disintegrin and metalloprotease domain-containing protein 10 (ADAM10)	Expressed in neuroepithelial regions and differentiating gray matter	Various transmembrane proteins such as APP, n-Cadherin, neurexin-1, neuroligin-1, and Cx3CL1 are substrates of ADAM10.; involved in learning and memory, and synaptic plasticity	[85,86]
ATP-binding cassette transporters (ABCA7)	Localized in the luminal domain of BBB endothelial cells and expressed in brain tissues	Conducts apolipoprotein-mediated transport of cholesterol and HDL affects Aβ clearance by phagocytosis	[87,88]
Beta1 adrenergic receptor (β-1 AR)	Member of g-protein-coupled receptor expressed in the brain and release adrenaline	Important role in learning and memory functions through TNFα signaling.	[11,89]
Bridging integrator 1 (BIN1)	Neuronal cells including pre- and post-synaptic compartments	Involved in Aβ peptide generation and tau spreading	[90,91,92]
CD2-associated protein	Present in endothelial cells	Involve in receptor-mediated endocytosis; regulate Aβ generation in neurons	[93]
Complement component Receptor 1 (CR1)	Type-1 transmembrane glycoprotein expressed on erythrocytes, and all blood cell types, CD4+ T cells follicular dendritic cells, and glomerular podocytes	Binds to C3b, a cofactor, and removes Aβ1–42 from the brain as well as from the circulatory system	[11,94]
Inositol-requiring protein I (IRE1)	Localized in the ER membrane, binds to misfolded proteins	Catalyzes the splicing of transcription factor box binding protein (XBP1) mRNA; degrades mRNAs of ER through RIDD under UPR	[95,96]
IL33	Expressed in astrocytes, oligodendrocytes, and in neurons binds to ST2 in microglia	Involved in synaptic plasticity and learning and memory; decreased IL33/ST2 signaling contributes to synaptic impairment	[97]
Leukocyte immunoglobulin-like receptor B2 (LILRB2)	Highly expressed in pyramidal neurons in visual cortex and hippocampus	PirB signaling is important for maintaining synapse density and plasticity; plays role in learning and memory	[63,98]
LDL receptor-related protein-1 (LRP1)	Abundantly expressed in the liver, neurons, astrocytes, and vasculatures in the brain	Binds with phosphatidylinositol-binding clathrin assembly (PICALM) to clear Aβ monomers, oligomers, and aggregates from the brain across the blood–brain barrier (BBB)	[99,100,101,102,103]
Microtubule-associated protein tau (MAPT)	Expressed in neurons, maintain microtubule structure in axons	Hyperphosphorylated and induced formation of tau aggregates and NFTs in AD	[11]
Phosphatidylinositol-binding clathrin assembly protein (PICALM)	Present in pre- and post-synaptic compartments and involve in regulating SV recycling	Involved in synaptic dysfunction in AD	[93]
Protein tyrosine kinase 2β (PTK2B)	Highly expressed in the hippocampus	Role in synaptic plasticity regulation and memory	[104,105]
Phospholipase D (PLD3)	Expressed in pyramidal neurons in the brain	A significant AD risk variant pA442A, altered microglia and lysosomal function	[106]
Presenilin (PSEN)	Mostly PSEN1- and PSEN2-encoded proteins expressed in brain	Involved in induced cleavage of APP results in Aβ peptide generation	[16]
Protein kinase RNA-like ER kinase (PERK)	Localized in the ER membrane, binds to misfolded proteins	Binds to eIF2α and Nrf2; potentially inhibits translation and restore ER homeostasis	[107,108]
Sortilin-related receptor 1 (SORL1)	Membrane bound protein containing VPS10 and the YWTD/EGF domain	Protein sorting and trafficking within the trans-Golgi network to the membrane and targets protein in the endosomal/lysosomal system, APP processing and trafficking, synapse formation and synaptic functions	[109,110]
Triggering receptor expressed on myeloid cell 2 (TREM2)	Expressed in the immune cells of myeloid origin	Activates downstream signaling in microglia	[58,111,112]

### 3.3. Signaling Pathways Associated with Aβ Production and Tau Phosphorylation

Metabolic dysfunction is a well-established symptom in AD, evidenced by glucose hypometabolism detectable even before the onset of AD symptoms. Individuals with insulin resistance, type 2 diabetes mellitus (T2D), hyperlipidemia, obesity, or other metabolic diseases are at a high risk of developing AD with aging [113]. Impaired insulin signaling has been linked to neuroinflammation and cognitive decline [114]. Insulin resistance is observed in brain tissues affected by AD, particularly in the hippocampus and cerebral cortex. In T2D, altered TNF-α/JNK signaling leads to insulin resistance in the hippocampus and cortex [115]. Phosphorylation of downstream signaling molecules like AKT, PI3K, and GSK-3β regulates the increase in Aβ production and tau phosphorylation in the brain. Another important regulator of GSK-3β is the mTOR pathway, which regulates neuronal growth, differentiation, and interconnectivity [9,34]. In AD models, mTOR activity was found to reduce with aging; thus, altered mTOR signaling might affect AD pathogenesis [116,117]. In the brain, insulin-responsive glucose transporters, specifically GLUT4 and GLUT8, are localized on the BBB and within neurons and glia. Chronic hyperinsulinemia leads to the downregulation of insulin transporters at the BBB, consequently reducing the amount of insulin that enters the brain [118]. Older adults with insulin resistance exhibited patterns of reduced brain glucose levels similar to those observed in AD, affecting the same brain regions. This supports the hypothesis that insulin resistance may ultimately contribute to the pathogenesis of AD [119].

Furthermore, sodium–glucose co-transporter-2 inhibitors (SGLT2), such as dapagliflozin and Canagliflozin, restore the mTOR pathway via nutrient-sensitive mechanism, resulting in activated glucose uptake and reduced blood glucose levels, potentially mitigating excitotoxicity in the neurons. This could lead to a reduction in tau phosphorylation and the accumulation of Aβ in AD models [120,121]. Additionally, caspases, Nrf2, and NF-κB indirectly influence this pathway. Moreover, CREB signaling is also implicated in AD pathology. CREB phosphorylation is altered in AD patients because of altered GSK3-β activity and PKA signaling [122]. CREB regulates neurotrophins, such as brain-derived neurotrophic factor (BDNF) and nerve growth factor (NGF), which are essential for cognitive processes [122]. There are reports that showed increased amyloid-β levels downregulate CREB signaling and reduce BDNF/NGF expression, leading to synaptic loss and cognitive dysfunction [123,124].

**Figure 2:**
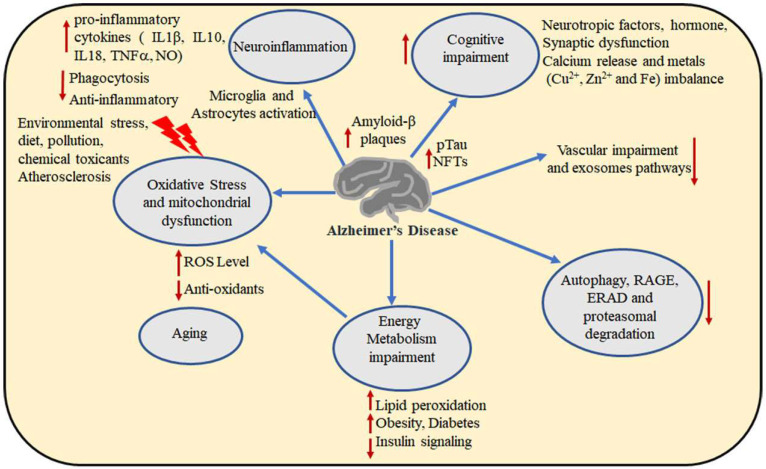
This image depicts various biological processes affected by AD. It illustrates increased amyloid-β plaques, tau hyperphosphorylation, elevated ROS levels, and impaired amyloid-β clearance pathways, along with metabolic abnormalities. Reduced degradation, heightened lipid oxidation and metabolite accumulation, mitochondrial dysfunction, and the aging process contribute to the progression of AD. Vascular impairments and defects in the exosome pathway heighten the risk of amyloid-β accumulation, while calcium release and metal dyshomeostasis exacerbate cognitive impairment and neuronal cell death.

Other pathways, including oxidative stress, mitochondrial OXPHOS, lipid metabolism, and autophagy failure, are also involved in AD pathology (Figure 2) [125,126]. Elevated ROS levels in the brain contribute to neuronal damage. The increased ROS result in elevated peroxidized (O^2−^) lipid and free radicals generated by mitochondrial dysfunction or metabolites in the brain remains unclear and requires more investigation. Studies on mice neuronal glia co-cultures indicate increased lipid droplets (LDs) in astrocytes depending on APOE gene expression in AD [127]. A GWAS identified that NDUFAF6 rs6982393, encoding an ADH-ubiquinone oxidoreductase important for mitochondrial assembly I, was linked to increased Aβ toxicity. Another gene variant rs11667768 is linked with phospholipase D3 (PLD3). PLD3 knockout mice showed an increase in lipid droplets in the brain and, subsequently, an increase in Aβ deposition [128]. GPR55, a G-protein-couple receptor implicated in glucose and energy homeostasis, and the RhoA/ROCK pathway were reported in Aβ1–42 in the hippocampus and frontal cortex in AD transgenic mice. In vivo pharmacological inhibition of GPR55 and RhoA/ROCK demonstrated neuroprotective effects, reducing apoptosis and oxidative stress induced by elevated Aβ levels in the mouse brain (Figure 2) [129,130].

GWASs focus on investigating potential risk factors associated with AD using clinical samples, leading to an increase in the number of loci and identification of new risk factors, SNPs, and mutations significantly associated with AD. However, these GWAS-identified genes are related to numerous pathways, necessitating systematic characterization to establish links among APP metabolism, tau function, and genetic risk factors for effective drug targeting and therapy. The TNF-α signaling pathway has been found to be highly associated with AD risk factors [131]. The inhibition of TNF-α signaling has shown a significant reduction in AD and tau pathology in vivo, including memory impairment, synaptic plasticity, and synapse loss in the brain [73,132]. CD42, a cell division cycle 42 protein and a Rho GTPase, has been identified as an important gene for AD progression in clinical hippocampus samples, with a *p*-value of 7.8 × 10^−6^ in DEG pathway enrichment analysis. CD44 is localized in both neuronal and glia cells and plays a role in neuroinflammation. A loss-of-function mutation in CD44 significantly exacerbates neurotoxicity associated with Aβ, thereby exacerbating cognition dysfunctions, NFT formation, and amyloid plaque accumulation [133,134]. RPH3A, a small G-protein involved in exocytosis of neurotransmitter release and synaptic vesicle traffic, is downregulated in vivo in AD mice [135]. Integrin β-5 (ITGB5), also known as CD18, is correlated with diabetic neuropathy and has been found to be associated with AD progression [136]. However, the underlying mechanisms need to be evaluated and confirmed in AD clinical samples.

### 3.4. Inflammatory Pathways Aid in Alzheimer’s Disease Progression

Inflammation stands as a primary acute response in various neurodegeneration diseases. In AD, the accumulation of amyloid plaques and NFTs within the neurons triggers the inflammatory signaling pathway. This activation leads to the release of either pro-inflammatory cytokines or anti-inflammatory cytokines within the brain tissues, particularly in microglia and astrocytes [131]. These cells engage in crosstalk, releasing different inflammatory cytokines and chemokines at the site of inflammation to aid in the clearance of Aβ plaques and aggregated proteins [131]. In AD, the accumulation of Aβ and tau fibrils in the brain and blood vessels compromise the function and integrity of the blood–brain barrier (BBB). This triggers the release of pro-inflammatory cytokines and activates myeloid cell-dependent neutrophil infiltration that results in the upregulation of adhesion molecules (e.g., VEGF) on brain endothelial cells [137]. Subsequently, neutrophils release neutrophil extracellular traps (NETs), exacerbating neuroinflammation and contributing to the accumulation of amyloid plaque and tau fibril tangles [138]. Moreover, this cascade of events obstructs cerebral blood flow (CBF), leading to cognitive dysfunction and dementia.

In studies using APP/PS1 and 5XFAD models of AD, the depletion of neutrophils with anti-Ly6G antibodies reduces the number of stalled capillaries, promoting revascularization in CBF and improving cognitive dysfunctions [138,139]. Additionally, blocking neutrophil trafficking and infiltration into the brain by inhibiting integrin LFA-1 reduces neurotoxicity and ameliorates memory deficits in AD mice. Furthermore, activated astrocytes also contribute to triggering inflammatory signaling, thereby secreting pro-inflammatory cytokines and chemokines that increase oxidative stress and, ultimately, neuronal cell death [140]. The transmembrane protein CD33, expressed on the microglia receptor, exhibits increased expression in AD. It modulates Aβ1–42 levels in microglia and monocytes, enhancing microglia phagocytosis of amyloid β in the AD brain [131]. APOE genes are implicated in neuroinflammation in both the microglia and astrocytes [141]. One GWAS identified APOE as a potential risk factor for AD, where APOE4 was strongly associated with AD progression and Aβ deposition compared with APOE2 and APOE3 in late-onset AD [25,141]. In APP, transgenic mice expression of APOE4 leads to an increase in fibrillar Aβ plaque burden compared with mice expressing APOE3 or APOE2. This increase has also been verified in APOE4 carrier individuals who show an increase in both vascular and parenchymal Aβ plaques [2,3,4]. In vivo studies using transgenic mice (P301S) showed that the expression of APOE4 induced neuronal atrophy and increased tau and p-tau levels in the brain, highlighting its significant role in AD pathology [25,84]. In vitro studies with APOE4 (iPSC)-derived glia revealed increased accumulation of unesterified cholesterol, triggering the expression of proinflammatory cytokines and chemokines, leading to neuronal cell death [83]. Triggering receptor expressed on myeloid cells 2 (TREM2), expressed in microglia, is reported as a risk factor modulating Aβ levels in AD [131,142,143]. Most AD-associated TREM2 variants are characterized by loss-of-function variants that lead to reduced protein expression or activity [144]. The most common variant, R47H TREM2, is linked to an increased risk of AD. The R47H TREM2 variant contributes to the disruption of synaptic connectivity and functions in the early stages of AD, preceding the onset of clinical symptoms [58]. Additionally, ATP-binding cassette transporter A7 expression was induced by Aβ1–42 in microglial cells in the brain, as revealed by GWASs [145].

Astrocyte activation and release of complement protein C3, binding to C3aR in neurons, induces neuronal damage [146]. Moreover, soluble C40 ligands from astrocytes bind to their cognate surface receptors in microglia, releasing TNF-α in AD, which promotes neuronal cell degradation [131]. For instance, in the hippocampus, TNF-mediated inflammation triggers necroptosis, a form of neuronal cell death driven by enhanced inflammation depending on TNFR1 signaling, was reported in the AD postmortem brain [147]. Specifically, the interaction between TNF and TNFR1 activates a phosphorylate cascade involving receptor-interacting protein kinase 1 (RIPK1). RIPK3 and mixed lineage kinase domain-like (MLKL) kinase induce inflammation-mediated necroptosis in the hippocampus in AD [148]. Importantly, targeting TNFR1 and RIPK1 has been shown to prevent neuronal cell death, suggesting a novel therapeutic target for AD treatment [149].

IL1β and IL-18 expression induced in glial cells during AD progression are regulated by NLRP3 inflammasome, which activates pyroptosis in glial cells and neurons. These activated cells release proinflammatory cytokines and chemokines, including TNF-α, IL1β, IL-6, and C3 ligand, which favors Aβ1–42 production and accumulation, ultimately leading to cell death [150]. The upregulation of TGF-β1, produced by SOD1 G93A reactive astrocytes, leads to cytoplasmic aggregation and disrupted autophagy in AD [151]. Several anti-inflammatory agents target NLRP3 inflammasome signaling. Various proteins including Neutrophil gelatinase-associated lipocalin (LCN2), progranulin (GRN), glia fibrillary acidic protein (GFAP), and TMEM106B have been identified in the cerebrospinal fluid of patients with AD and other types of dementia, such as amyotrophic lateral sclerosis, frontotemporal, dementia, and Parkinson’s disease, and are correlated with reactive astrocyte pathology [152,153]. Despite these findings, the role of reactive astrocytes and microglia in enhancing cytokine release, leading to increased NF-kβ activation and impaired autophagy in AD progression, warrants further investigation.

Microglia, the resident immune cells in the brain, play a critical role in phagocytosis and the autophagic clearance of cellular waste and toxic protein aggregates. In AD, microglial activation leads to cytokine secretion and increased phagocytosis activity. The efficacy of microglia responses to stress stimuli and their phagocytic functions relies on functional lysosomal regulatory circuits [154]. Studies have demonstrated that defective lysosomal acidification in microglia results in impaired lysosomal function, which in turn enhances the release of inflammatory cytokines and induces neuronal cell death via mechanisms such as necroptosis [155]. This impairment in lysosomal acidification compromises microglia phagocytosis and involves various cellular modulations, including presenilin modifications, cytokine and inflammatory stimulation, and mitochondrial dysfunction, whose mechanism remains unclear and needs to be investigated [156]. Presenilin 1 (PSEN1, PS1) and Presenilin 2 (PSEN2, PS2) are essential for APP cleavage and Aβ generation. PS1 is particularly important for microglia activation and cytokine release [157], with its phosphorylation being crucial for microglia activation and lysosomal acidification. In contrast, PS2 N1411 mutant mice exhibit increased cytokine release and activated microglia, highlighting its role in Aβ phagocytosis and its significance in AD pathology [158]. Another key factor in lysosomal acidification is the activity of vacuolar (H+)-ATPase, which is linked with mitochondrial dysfunctions and increased ROS. The interplay between lysosome and mitochondria dysfunction in AD pathology is well documented, though the precise mechanism underlying these deficits remains incompletely understood and warrants further detailed investigation [159,160].

Restoring lysosomal acidification has been shown to mitigate microglial impairment and improve lysosomal functions. Various small molecules have been employed to achieve this, such as C381 and EN6, which act on the V-ATPase complex to maintain lysosomal pH [161]. Other modifiers include SF-22 and its analog, which targets TRP channel protein to regulate lysosomal pH [162]. Tetrandrine, which inhibits TPC2, facilitates lysosomal acidification and enhances the autophagic degradation of pathogenic tau aggregates [162,163]. Curcumin analog C1 and PF11 promote lysosomal biogenesis and luminal acidification [164]. Additionally, mTOR inhibitors such as OS1–027 and PP242 improve autophagic function and lysosomal acidification by increasing cathepsin D activity [165]. Nanoparticle-based compounds have also shown efficacy in restoring lysosomal acidification under pathological conditions. These include poly (lactic-co-glycolic acid) (PLGA) nanoparticles (NPs), acidic NPs (ACNPs), photo-activated NPs (PaNPs), and acidic nucleolipid nanoemulsions (NL-NEs) [166,167,168]. Further optimization is needed for clinical application, including the development of BBB-penetrating peptides on the surface of NPs and the selection of suitable acids that are well metabolized in the body with minimal side effects [169].

Astrocytes play a crucial role in maintaining energy levels through different ion channels, which have been found to be dysregulated in patients with LOAD [170,171]. Numerous studies indicate that metabolic dysfunction exacerbates Aβ production and impairs the clearance of Aβ plaques and tau neurofibrillary tangles because of compromised degradative pathways. Importantly, metabolic dysfunction in astrocytes leads to oxidative stress and neuroinflammation, contributing to Aβ pathology [125,172]. Additionally, microglial activation exacerbates inflammation and tau seeding in AD mice, selectively increasing NF-κB signaling in microglia, which induces inflammation and tau-mediated synaptic loss. In vivo, it has been observed that microglia support the spread of tau by taking up and breaking down the seed-component form of tau [173]. Inhibition of microglial proliferation has been shown to attenuate tau-induced neurodegeneration and cognitive deficits [174]. In LOAD, increased Aβ production is modulated by NF-κB signaling in the AD brain. In vitro studies have demonstrated that inhibiting NF-κB signaling in microglia via p65 deacetylation reduces Aβ production, suggesting NF-κB as a promising target in AD [175,176]. These observations indicate a crosstalk between glial cells and astrocytes in modulating Aβ levels in early- and late-onset AD, warranting further investigation in AD models.

## 4. Clearance and Degradation Pathways Implicated in AD

### 4.1. Receptor for Advance Glycation End Product-Mediated Aβ Clearance

The Receptor for Advance Glycation End Product (RAGE) is a multiligand receptor belonging to the immunoglobulin superfamily. It is involved in neuronal cell migration and differentiation during development, is susceptible to perturbation by Aβ, and plays a role in the inflammatory response [177]. RAGE exists in two isoforms including a full-length-membrane bound form (mRAGE) and a soluble form (sRAGE) lacking both the transmembrane and cytosolic domains [178,179]. Research indicates that the expression of RAGE changes in various brain cell types, including neurons, glia, astrocytes, and microglia. RAGE plays a crucial role as a transporter by regulating the influx of circulating Aβ into the brain across the blood–brain barrier (BBB), while the efflux of brain-derived Aβ into the circulation from the BBB is facilitated by LRP1 and P-glycoprotein [180,181]. The activation of RAGE in microglia also triggers the release of pro-inflammatory cytokines, such as IL-1β and TNF-α, contributing to neuronal impairment during the progression of AD. The binding of Aβ to RAGE in neurons and microglia leads to oxidative stress and inflammation, resulting in cellular perturbation and decreased learning ability in AD mouse models [182]. Moreover, p38 MAPK activation is essential for RAGE-dependent NF-κB activation, induction of target gene expression, and secretion of pro-inflammatory cytokines from monocytes.

NF-κB, an oxidant-sensitive transcription factor, exacerbates the pro-inflammatory response by regulating gene expression in RAGE ligands associated with aging, vascular pathology, inflammation, and hyperglycemia, thereby generating oxidative stress. Thus, the RAGE signaling axis, involving p38MAPK activation in neuronal and non-neuronal cells, contributes to the development of inflammatory responses and neuronal perturbation, particularly in response to increased Aβ accumulation during AD progression. In vivo studies have demonstrated that perturbation of p38MAPK leads to a reduction in Aβ-induced cytokine production and neuronal cell death in mouse models [183,184]. Conversely, the inhibition of RAGE significantly ameliorates Aβ-mediated sustained neuronal and microglial stress, consequently enhancing cognitive function in AD mice [185]. Despite these promising findings, there is currently no clinical RAGE inhibitor available for AD patients. Therefore, RAGE stands out as a potential therapeutic target for reducing the burden of amyloid-β in the brain, mitigating neuroinflammation, and preserving cognitive functions.

### 4.2. Endoplasmic Reticulum-Associated Aβ Clearance

Alzheimer’s disease (AD) involves alterations in endoplasmic reticulum (ER) functions and associated proteins, impacting disease progression and disease etiology. Amyloid-β plaque formation is linked to improper protein folding and aggregation of Aβ peptides. Unfolded Protein Response (UPR) triggers endoplasmic reticulum (ER) stress and ER-associated degradation (ERAD) implicated in AD pathology. The ER lumen, where protein folding occurs, relies on chaperones like BiP/GRP78, protein disulfide isomerase, calnexin, and calreticulin for proper folding and glycosylation of newly synthesized protein [186]. Furthermore, folded proteins proceed to the Golgi apparatus by vesicles, while misfolded proteins are either refolded or directed to degradation via ERAD [187]. ERAD prevents misfolded protein accumulation by translocating peptides to lysosomes for degradation via the ubiquitin–proteasome system or autophagy as well as lysosomal pathways to eliminate misfolded proteins [187,188]. Several studies indicated that mutant PS1 (A246E) and dE9 (deletion of exon 9) induce ER stress/UPR in 3xTg-AD mice and neuronal cell line SK-N-SH cells more than WT PS1, implicating UPR in AD and suggesting it as a treatment target. This suggests that ER stress and UPR are implicated in AD and can be a novel target for AD treatment.

ER stress triggers a complex network of signaling events and cellular processes involved in the degradation of unfolded or misfolded protein through the Ubiquitous Proteasomal system (UPS). Under ER stress inositol-requiring protein I (IRE1), a type I transmembrane protein, catalyzes the processing of X-box binding protein 1 (XBP1), leading to the activation of UPR-targeted genes that regulate the degradation of APP in steady-state conditions through the ERAD pathway [182]. Clinical AD brain samples show increased expression of ER stress and UPR-related genes like XBP1, CANX, PDIA3, PDIA6, HSPA5 (BiP/GRP78), and DNAJC3 at the mRNA level though protein levels vary across Braak stages 0-VI [189,190]. GWASs support ER genes like protein kinase RNA-like ER kinase (PERK) and inositol-requiring protein I (IRE1), a type I transmembrane protein, as a risk factor for AD [108,191]. The ablation of IRE1 in the mouse central nervous system reduces Aβ and amyloid proteins, attenuates astrocyte proliferation, and improves synaptic function [96]. Downstream target X-box binding protein (XBP1) degrades mRNA, rRNAs, and microRNAs, reducing UPR in ER. Inducing XBP1 expression in the AD mice hippocampus reduces Aβ levels and improves synaptic plasticity and memory function [192].

Studies have shown that the eIF2α kinases, PERK, GCN2 (general control non-derepressible 2), and PKR (double-stranded RNA-dependent protein kinase), play crucial roles in deficits observed in brain mRNA translation, synaptic plasticity, and memory in APP/PS1 mouse. In vitro, Aβ elevates eIF2α levels, and the knockout of either PKR or ATF4 renders neurons less susceptible to Aβ-induced toxicity. Consequently, the inhibition of PERK/eIF2α signaling reduces amyloid plaque formation and restores synaptic functions [107,193]. UPR activation ultimately restores mitochondrial functions, reducing Aβ toxicity and enhancing neuronal survival, thus indicating ER-associated protein degradation as a promising target for AD treatment. Further molecular investigation is essential to elucidate how ERAD regulates amyloid-β levels accurately in AD.

### 4.3. Autophagy-Mediated Amyloid-β Clearance in Alzheimer’s Disease

The progression of AD involves enhanced production of Aβ because of mutations in *APP* and *PS1/2* genes in familial AD cases or dysfunction of Aβ clearance pathways in sporadic AD cases. Clearance mechanisms encompass processes like phagocytosis, endocytosis, and enzymatic degradation by neprilysin, insulin-degrading enzymes, and matrix metalloproteinases. Various brain cells, including microglia, perivascular macrophages, and astrocytes, participate in Aβ clearance processes [14]. The in-depth clearance mechanism shows that Aβ in various brain cells effluxes from the brain to the periphery. Aβ effluxes are normally mediated via its receptors on the brain endothelium, mainly mediated by LDL receptor-related protein-1 (LRP-1). LDL receptor proteins bind with phosphatidylinositol-binding clathrin assembly (PICALM) to clear Aβ monomers, oligomers, and aggregates from the brain across the blood–brain barrier (BBB) [99]. LDL receptor binding protein 2 also aids Aβ trafficking across the BBB via binding with apolipoprotein J [102]. In mice, LRP1 knockout is embryonically lethal. Therefore, studying the role of LRPI in embryonic stages in mice is very critical. However, specific conditional knockout of LRP1 using Cre-recombinase in mice leads to reduced Aβ efflux from the brain, potentially contributing to the progression of AD [101]. Furthermore, LRP1 modulates the transport and clearance pathway involved in the uptake and clearance of Aβ from the brain [194]. Genetic evidence indicates that MEOX2, a homeobox gene, regulates LRP1 expression at the BBB [195] and potentially links to neurovascular dysfunction in AD. However, modulation of the MEOX2 gene in APP/PS1 mice did not show a significant effect on plaque deposition [196]. Therefore, further investigation is necessary to provide deep insight into LRP1 activity in AD that can be utilized for AD prevention. Furthermore, the ubiquitinated proteasome system, implicated in Aβ clearance, is impaired because of induced levels of Aβ and tau hyperphosphorylation, leading to increased amyloid plaques and NFTs in AD. Aβ plaques resist proteolytic degradation, and the role of the ubiquitin–proteasome system in Aβ clearance remains unclear, necessitating further investigation to reduce Aβ burden [14].

Autophagy is a crucial pathway for the degradation and clearance of aggregated Aβ proteins, plaques, and NFTs. Autophagy exhibits a complex enzyme system, containing acidic proteases and acid hydrolases, which form autophagosomes and ultimately fuse with late endosomes for the lysis of aggregated or misfolded proteins from the brain. The dysregulation of autophagy is attributed to the defective transport of autophagic vesicles from the axonal terminal to the soma, impairing lysosomal degradation and leading to the accumulation of immature autophagosomes and dystrophic neurites. This results in elevated levels of Aβ1–42 level in AD [197]. With aging, the autophagic pathway becomes impaired, as observed in human and animal models, such as mice, flies, zebrafish, and Xenopus, which showed increased extracellular Aβ levels due to high Aβ secretion and impaired exocytosis. Impaired exocytosis leads to the accumulation of Aβ in intracellular vesicles and subsequent impaired memory in AD mice [198,199]. Depletion of essential autophagy genes, such as *atg5* and *atg7*, in mice results in progressive neurodegeneration and accumulation of aggregated proteins in the brain [200,201]. The expression of *atg5*, *atg7*, and *beclin-1* declines with aging, affecting the lysosomal-mediated degradation of Aβ and NFTs plaques in the brain and contributing to late-onset neurodegeneration, including AD [202,203].

In 3XTg-AD mice, the expression of the autophagy-related genes *beclin-1* and *p62* decreases with AD progression compared with normal individuals [204,205]. In *ApoE4* transgenic mice, elevated levels of Aβ42 in the lysosomes lead to neuronal cell death in the hippocampus [100]. Furthermore, sirtuins play a crucial role in triggering the autophagy pathway. SIRT2 exerts a negative influence on the autophagy process. Induced expression of SIRT2 decreases autophagy in the brain by deacetylation of FOXO1, a member of the Forkhead O family of proteins. Various studies indicated that mitochondrial dysfunction triggers SIRT2 activation, resulting in microtubule (MT) disruption and impairment of the autophagic–lysosomal pathway in AD. SIRT2 inhibition or knockdown can prevent dysregulation of the autophagy–lysosomal pathway and subsequent toxicity caused by the accumulation of damaged mitochondria and Aβ peptides [206,207]. Therefore, SIRT2 could be a promising candidate for Aβ clearance via the induction of autophagy. Rapamycin, an mTOR pathway inhibitor, activates autophagy and reduces amyloid-β deposition in EOAD mice models; however, no effects are observed in LOAD. This raises an ambiguity in the role of mTOR-mediated autophagy activation in AD that warrants further investigation [193].

**Figure 3:**
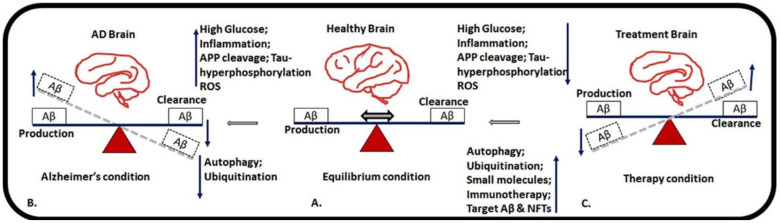
This image represents different conditions of AD in normal (**A**), AD (**B**), and treatment conditions (**C**). The image illustrates the imbalance in Aβ formation and clearance of Aβ aggregates in human brains over the course of aging. The equilibrium state depicts a balance between Aβ production and Aβ clearance. However, in the AD condition, there is an increase in Aβ production and decreased Aβ clearance, which contribute to disease progression. AD treatment aims to restore brain functions by reducing Aβ aggregate formation and inducing Aβ clearance via various treatment strategies such as induced autophagy, ubiquitination, immunotherapy, small molecule therapy, and degradation of Aβ and NFTs.

It remains unclear whether the accumulation of Aβ in LOAD is due to a lack of an efficient clearing system or enhanced production of Aβ (Figure 3). Genetic ablation of autophagy components leads to reduced Aβ secretion and decreased accumulation of intracellular Aβ, exacerbating neurodegeneration [198]. Thus, autophagy plays a dual role either as pro-survival or pro-death functions and depends on the Aβ level in neurons, necessitating further molecular investigations to gain insights into the underlying mechanisms.

## 5. Conclusions and Future Prospects

In this review, various factors and molecular targets have been highlighted to gain more insight into therapeutic targets in AD. Numerous studies have identified various genetic risk factors, mutations, and changes in cerebrospinal fluid (CSF) and plasma-based biomarkers associated with AD pathology. Despite these findings, the exact cause of AD remains elusive [208]. While a plethora of genes and proteins have been identified as biomarkers in different brain regions, they have not yet provided a complete cure for AD, only offering ways to slow its progression [209]. Consequently, no approved therapy exists to cure AD fully. However, early detection is crucial for effective treatment. Genome-wide association studies (GWASs) hold promise for identifying precise targets for early detection, either through neuroimaging or plasma-based biomarkers. These targets could aid in developing potential therapies to combat AD.

**Figure 4:**
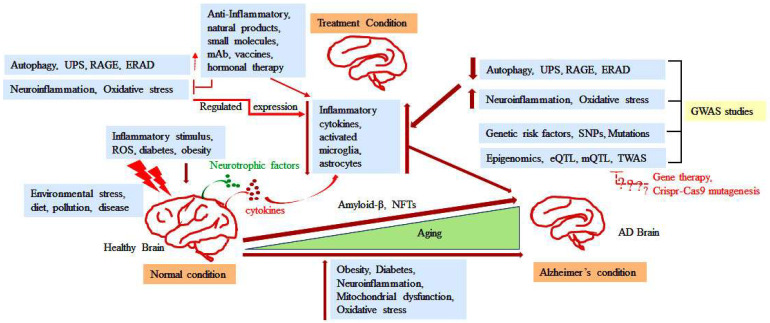
This image illustrates various factors associated with AD and highlights the impact of several stressors on a healthy brain and the subsequent consequences that lead to disease progression. GWASs and related genetic factors, as well as specific pathways, are shown to ameliorate disease conditions. Targeting different pathways can slow disease progression and improve cognitive functions. However, genetic manipulations and gene therapy for AD treatment still require extensive in vivo testing. This figure also depicts strategies to restore brain functions by reducing Aβ aggregates and promoting Aβ clearance through induced autophagy, UPS, RAGE, and ERAD. Additionally, immunotherapy and small molecule therapies are illustrated as approaches to reduce inflammation and maintain energy homeostasis, ultimately slowing AD progression. Red ‘?’ shows the effects are remains exclusive.

Currently, several drugs have undergone Phase II clinical trials, including monoclonal antibodies (mAbs), which have emerged as promising disease-modifying agents. Some of these include Gosuranemab, Tilavonemab, Semorinemab, Zagotenemab, Aducanumab [210], Lecanemab [211], and anti-tau antibodies, which have shown significant improvement in patients at early stages [20]. However, vaccines targeting the tau protein, such as the anti-tau vaccine (AADvac1) and ACI-35, are yet to demonstrate therapeutic outcomes [212,213]. Additionally, pharmacological treatments such as donepezil, galantamine, rivastigmine, and memantine are currently available drugs that mostly inhibit acetylcholinesterase and activate NMDA receptors, offering symptomatic relief in AD [20,212,214]. Moreover, the JAK2 inhibitor, TG101209, mitigated IFNγ-induced alterations in cultured microglia and microglia derived from APP/PS1 mice [215]. Similarly, the RAF inhibitor sorafenib reversed memory impairment and decreased the expression of APP, Cox-2, and iNOS in the brain of an AD transgenic mouse model, highlighting the potential of targeting RAF1 [215]. These findings suggest that JAK2 and RAF1 are promising therapeutic targets for AD and strategies aimed at reducing neuroinflammation. The limitations and potential side effects of monoclonal antibodies targeting Aβ and tau fibrils in AD patients raise significant safety concerns. These therapeutics primarily manage symptoms and delay the onset of AD, but they are insufficient for a complete cure (Figure 4). Therefore, it is imperative to explore new targets that address the underlying causes of AD rather than merely alleviating symptoms and to develop innovative drug therapies. In this context, GWAS data can elucidate the functional significance of gene mutations and SNPs in AD patients. Further validation of these risk factors genes and associated SNPs in animal models and clinical samples from AD patients will aid in identifying targets related to autophagy and ERAD for preventing AD progression. Most studies have focused on inhibiting Aβ production through the inhibition of γ-secretase cleavage rather than Aβ degradation or clearance. Therefore, further investigations are warranted to find novel pathways and molecular mechanisms that reduce Aβ levels either by APP processing enzymes or other multifunctional enzyme systems, including different degradation pathways, to prevent amyloidosis in LOAD.

Prominent genetic risk factors associated with Alzheimer’s disease (AD), along with numerous molecular factors, play a crucial role in the early detection of AD risk genes. Additionally, genes exhibiting common variants in AD and other types of dementia and neurodegeneration may establish an earlier link with AD progression, warranting in-depth investigation to determine the precise time point markers at which they intersect. Autophagy and ERAD pathways emerge as potential therapeutic avenues for managing neurodegenerative diseases, including AD. Understanding the specific proteolytic processes involved in the processing of proteins like APP and tau is paramount for unraveling the molecular mechanisms underlying AD.

In AD, metabolic dysregulation plays a critical role in disease progression and synaptic dysfunction. T2D, which is characterized by hyperglycemia and IR, affects brain glucose levels and promotes the accumulation of Aβ in the brain [216]. T2D is recognized as a risk factor for the progression of AD. However, the mechanism by which IR influences glucose metabolism, including its effects on memory and synapse plasticity, remains inadequately explored. Impaired glucose metabolism and the resulting formation of Aβ plaques and NFTs induce oxidative stress, leading to elevated intracellular free Ca^2+^ levels and triggering a cascade of detrimental effects that culminate in neuronal death [217]. Therefore, maintaining optimal brain glucose metabolism could mitigate oxidative damage, reduce the level of reactive oxygen species (ROS) and reactive nitrogen species (RNS), and protect the brain from adverse effects, thereby slowing disease progression. Continued research into glucose metabolism, oxidative stress, and mitochondrial dysfunction is essential for a more comprehensive understanding of AD pathogenesis and progression. These studies will also enhance the ability to monitor the therapeutic efficacy of novel drugs and small molecules targeting Aβ production and synaptic dysfunction, ultimately aiming to prevent neuronal cell death [218].

Furthermore, defective lysosomal acidification plays a crucial role in AD pathogenesis and progression. Impaired functions of microglia and astrocytes contribute to the accumulation and insufficient clearance of Aβ plaques and NFTs, leading to enhanced neuroinflammation and ultimately neuronal cell death. Lysosomal acidification defects and impaired glial functions occur early in the disease process, contributing to subsequent neuronal dysfunction. Therefore, the early detection of lysosomal acidification dysfunction and the development of therapeutic agents to restore lysosomal function are essential for effective AD therapy. Non-invasive, real-time detection methods would significantly advance this therapeutic approach [161,169]. Recent reports indicate that autolysosome acidification declines in neurons well before extracellular amyloid deposition, characterized by significantly reduced V-ATPase activity and the accumulation of Aβ/APP-βCTF within enlarged deacidified autolysosomes. These profuse Aβ-positive autophagic vacuoles (AVs) cluster into large membrane blebs, forming flower-like perikaryal rosettes known as PANTHOS, which are observed in AD brains. These AVs merge into perinuclear networks of membrane tubules where fibrillar β-amyloid accumulates intraluminal. This process leads to lysosomal membrane permeabilization, cathepsin release, and lysosomal cell death, followed by microglial invasion and phagocytosis. Additionally, neurons exhibiting PANTHOS are identified as the primary source of senile plaques in APP-associated AD models. This hypothesis suggests that the early detection of these molecular markers could serve as early diagnosis biomarkers for Alzheimer’s disease and aid in the development of therapeutic agents [219].

Moreover, the role of autophagy and ERAD in neurodegenerative diseases remains an active and evolving field of study. Optimizing pharmacological agents, such as small molecules and nanomedicines, for clinical application is critical. This includes enhancing their properties for better efficacy, bioavailability, and safety in therapeutic use [169]. Notably, these research strategies may provide deeper insights into the roles of specific proteases within various cellular compartments, aiding in the development of targeted therapies to inhibit plaque formation and prevent the development of NFTs. Future research should prioritize obtaining more population-based GWAS data from diverse cohorts and clinical samples. This approach will be crucial for identifying novel AD risk factors and potential targets for innovative drug therapies and treatment strategies.

## Abbreviations

AD	Alzheimer’s disease
APP	amyloid precursor protein
APH1	anterior pharynx defective 1
BACE1	β-site APP-cleaving enzyme-1
BBB	blood–brain barrier
CSF	cerebrospinal fluid
EOAD	Early-Onset Alzheimer’s Disease
ER	endoplasmic reticulum
ERAD	ER-associated protein degradation
eQTL	expression quantitative trait loci
eTWASs	expression transcriptome-wide association studies
FTD	familial frontotemporal dementia
GWASs	genome-wide association studies
IGAP	International Genomics of Alzheimer’s Project
LOAD	late-onset Alzheimer’s disease
LRP-1	LDL receptor-related protein-1
LD	linkage disequilibrium
MMPs	matrix metalloproteinases
MAPT	microtubule-associated protein
mQTL	methylation QTL
NFTs	neurofibrillary tangles
PICALM	phosphatidylinositol-binding clathrin assembly
RAGE	Receptor for Advanced Glycation End Product
SRP14	Single Recognition Particle 14
α7nAChRs	α7-Nicotinic Acetylcholine Receptors
SNPs	single nucleotide polymorphisms
TNF	tumor necrosis factor
TREM2	triggering receptor expressed on myeloid cells 2
UPR	unfolded protein response
UPS	ubiquitous proteasomal system
XBP1	X-box binding protein 1

## References

[1] Breijyeh Z., Karaman R. (2020). Comprehensive review on alzheimer’s disease: Causes and treatment. Molecules.

[2] Knopman D.S., Amieva H., Petersen R.C., Chételat G., Holtzman D.M., Hyman B.T., Nixon R.A., Jones D.T. (2021). Alzheimer disease. Nat. Rev. Dis. Primers.

[3] Wang Y., Mandelkow E. (2016). Tau in physiology and pathology. Nat. Rev. Neurosci..

[4] Zhou L., McInnes J., Wierda K., Holt M., Herrmann A.G., Jackson R.J., Wang Y.-C., Swerts J., Beyens J., Miskiewicz K. (2017). Tau association with synaptic vesicles causes presynaptic dysfunction. Nat. Commun..

[5] Better M.A. (2024). 2024 alzheimer’s disease facts and figures. Alzheimer’s Dement..

[6] Hanger D.P., Byers H.L., Wray S., Leung K.-Y., Saxton M.J., Seereeram A., Reynolds C.H., Ward M.A., Anderton B.H. (2007). Novel phosphorylation sites in tau from alzheimer brain support a role for casein kinase 1 in disease pathogenesis. J. Biol. Chem..

[7] Ittner A., Chua S.W., Bertz J., Volkerling A., Van Der Hoven J., Gladbach A., Przybyla M., Bi M., Van Hummel A., Stevens C.H. (2016). Site-specific phosphorylation of tau inhibits amyloid-β toxicity in alzheimer’s mice. Science.

[8] Andrade-Guerrero J., Santiago-Balmaseda A., Jeronimo-Aguilar P., Vargas-Rodríguez I., Cadena-Suárez A.R., Sánchez-Garibay C., Pozo-Molina G., Méndez-Catalá C.F., Cardenas-Aguayo M.-d.-C., Diaz-Cintra S. (2023). Alzheimer’s disease: An updated overview of its genetics. Int. J. Mol. Sci..

[9] Kim J., Basak J.M., Holtzman D.M. (2009). The role of apolipoprotein e in alzheimer’s disease. Neuron.

[10] Liu C.-C., Zhao N., Fu Y., Wang N., Linares C., Tsai C.-W., Bu G. (2017). Apoe4 accelerates early seeding of amyloid pathology. Neuron.

[11] Nystuen K.L., McNamee S.M., Akula M., Holton K.M., DeAngelis M.M., Haider N.B. (2024). Alzheimer’s disease: Models and molecular mechanisms informing disease and treatments. Bioengineering.

[12] Jansen I.E., Savage J.E., Watanabe K., Bryois J., Williams D.M., Steinberg S., Sealock J., Karlsson I.K., Hägg S., Athanasiu L. (2019). Genome-wide meta-analysis identifies new loci and functional pathways influencing alzheimer’s disease risk. Nat. Genet..

[13] Takatori S., Wang W., Iguchi A., Tomita T. (2019). Genetic risk factors for alzheimer disease: Emerging roles of microglia in disease pathomechanisms. Reviews on Biomarker Studies in Psychiatric and Neurodegenerative Disorders.

[14] J Baranello R., L Bharani K., Padmaraju V., Chopra N., K Lahiri D., H Greig N., A Pappolla M., Sambamurti K. (2015). Amyloid-beta protein clearance and degradation (abcd) pathways and their role in alzheimer’s disease. Curr. Alzheimer Res..

[15] Vassar R., Kovacs D.M., Yan R., Wong P.C. (2009). The β-secretase enzyme bace in health and alzheimer’s disease: Regulation, cell biology, function, and therapeutic potential. J. Neurosci..

[16] Kimberly W.T., LaVoie M.J., Ostaszewski B.L., Ye W., Wolfe M.S., Selkoe D.J. (2003). Γ-secretase is a membrane protein complex comprised of presenilin, nicastrin, aph-1, and pen-2. Proc. Natl. Acad. Sci. USA.

[17] Hampel H., Hardy J., Blennow K., Chen C., Perry G., Kim S.H., Villemagne V.L., Aisen P., Vendruscolo M., Iwatsubo T. (2021). The amyloid-β pathway in alzheimer’s disease. Mol. Psychiatry.

[18] Li C., Götz J. (2017). Tau-based therapies in neurodegeneration: Opportunities and challenges. Nat. Rev. Drug Discov..

[19] Nozal V., Martinez A. (2019). Tau tubulin kinase 1 (ttbk1), a new player in the fight against neurodegenerative diseases. Eur. J. Med. Chem..

[20] Monteiro A.R., Barbosa D.J., Remião F., Silva R. (2023). Alzheimer’s disease: Insights and new prospects in disease pathophysiology, biomarkers and disease-modifying drugs. Biochem. Pharmacol..

[21] Li Z., Shue F., Zhao N., Shinohara M., Bu G. (2020). Apoe2: Protective mechanism and therapeutic implications for alzheimer’s disease. Mol. Neurodegener..

[22] Shinohara M., Kanekiyo T., Tachibana M., Kurti A., Shinohara M., Fu Y., Zhao J., Han X., Sullivan P.M., Rebeck G.W. (2020). Apoe2 is associated with longevity independent of alzheimer’s disease. Elife.

[23] Arboleda-Velasquez J.F., Lopera F., O’Hare M., Delgado-Tirado S., Marino C., Chmielewska N., Saez-Torres K.L., Amarnani D., Schultz A.P., Sperling R.A. (2019). Resistance to autosomal dominant alzheimer’s disease in an apoe3 christchurch homozygote: A case report. Nat. Med..

[24] Chemparathy A., Le Guen Y., Chen S., Lee E.-G., Leong L., Gorzynski J.E., Jensen T.D., Ferrasse A., Xu G., Xiang H. (2023). Apoe loss-of-function variants: Compatible with longevity and associated with resistance to alzheimer’s disease pathology. Neuron.

[25] Parhizkar S., Holtzman D.M. (2022). Apoe mediated neuroinflammation and neurodegeneration in alzheimer’s disease. Semin. Immunol..

[26] Homann J., Osburg T., Ohlei O., Dobricic V., Deecke L., Bos I., Vandenberghe R., Gabel S., Scheltens P., Teunissen C.E. (2022). Genome-wide association study of alzheimer’s disease brain imaging biomarkers and neuropsychological phenotypes in the european medical information framework for alzheimer’s disease multimodal biomarker discovery dataset. Front. Aging Neurosci..

[27] Barone E., Di Domenico F., Mancuso C., Butterfield D.A. (2014). The janus face of the heme oxygenase/biliverdin reductase system in alzheimer disease: It’s time for reconciliation. Neurobiol. Dis..

[28] Heath L., Earls J.C., Magis A.T., Kornilov S.A., Lovejoy J.C., Funk C.C., Rappaport N., Logsdon B.A., Mangravite L.M., Kunkle B.W. (2022). Manifestations of alzheimer’s disease genetic risk in the blood are evident in a multiomic analysis in healthy adults aged 18 to 90. Sci. Rep..

[29] Litvinchuk A., Suh J.H., Guo J.L., Lin K., Davis S.S., Bien-Ly N., Tycksen E., Tabor G.T., Serrano J.R., Manis M. (2024). Amelioration of tau and apoe4-linked glial lipid accumulation and neurodegeneration with an lxr agonist. Neuron.

[30] Tracy T.E., Gan L. (2018). Tau-mediated synaptic and neuronal dysfunction in neurodegenerative disease. Curr. Opin. Neurobiol..

[31] Maday S., Twelvetrees A.E., Moughamian A.J., Holzbaur E.L. (2014). Axonal transport: Cargo-specific mechanisms of motility and regulation. Neuron.

[32] Sherman M.A., LaCroix M., Amar F., Larson M.E., Forster C., Aguzzi A., Bennett D.A., Ramsden M., Lesné S.E. (2016). Soluble conformers of aβ and tau alter selective proteins governing axonal transport. J. Neurosci..

[33] Abyadeh M., Gupta V., Paulo J.A., Mahmoudabad A.G., Shadfar S., Mirshahvaladi S., Gupta V., Nguyen C.T., Finkelstein D.I., You Y. (2024). Amyloid-beta and tau protein beyond alzheimer’s disease. Neural Regen. Res..

[34] Wang J., Gu B.J., Masters C.L., Wang Y.-J. (2017). A systemic view of alzheimer disease—Insights from amyloid-β metabolism beyond the brain. Nat. Rev. Neurol..

[35] Fá M., Puzzo D., Piacentini R., Staniszewski A., Zhang H., Baltrons M.A., Li Puma D.D., Chatterjee I., Li J., Saeed F. (2016). Extracellular tau oligomers produce an immediate impairment of ltp and memory. Sci. Rep..

[36] Brand A.L., Lawler P.E., Bollinger J.G., Li Y., Schindler S.E., Li M., Lopez S., Ovod V., Nakamura A., Shaw L.M. (2022). The performance of plasma amyloid beta measurements in identifying amyloid plaques in alzheimer’s disease: A literature review. Alzheimer’s Res. Ther..

[37] Leuzy A., Janelidze S., Mattsson-Carlgren N., Palmqvist S., Jacobs D., Cicognola C., Stomrud E., Vanmechelen E., Dage J.L., Hansson O. (2021). Comparing the clinical utility and diagnostic performance of csf p-tau181, p-tau217, and p-tau231 assays. Neurology.

[38] Suárez-Calvet M., Karikari T.K., Ashton N.J., Lantero Rodriguez J., Milà-Alomà M., Gispert J.D., Salvadó G., Minguillon C., Fauria K., Shekari M. (2020). Novel tau biomarkers phosphorylated at t181, t217 or t231 rise in the initial stages of the preclinical alzheimer’s continuum when only subtle changes in aβ pathology are detected. EMBO Mol. Med..

[39] Ashton N.J., Benedet A.L., Pascoal T.A., Karikari T.K., Lantero-Rodriguez J., Brum W.S., Mathotaarachchi S., Therriault J., Savard M., Chamoun M. (2022). Cerebrospinal fluid p-tau231 as an early indicator of emerging pathology in alzheimer’s disease. EBioMedicine.

[40] Dolphin H., Dyer A.H., Morrison L., Shenkin S.D., Welsh T., Kennelly S.P. (2024). New horizons in the diagnosis and management of alzheimer’s disease in older adults. Age Ageing.

[41] Thijssen E.H., La Joie R., Wolf A., Strom A., Wang P., Iaccarino L., Bourakova V., Cobigo Y., Heuer H., Spina S. (2020). Diagnostic value of plasma phosphorylated tau181 in alzheimer’s disease and frontotemporal lobar degeneration. Nat. Med..

[42] Janelidze S., Mattsson N., Palmqvist S., Smith R., Beach T.G., Serrano G.E., Chai X., Proctor N.K., Eichenlaub U., Zetterberg H. (2020). Plasma p-tau181 in alzheimer’s disease: Relationship to other biomarkers, differential diagnosis, neuropathology and longitudinal progression to alzheimer’s dementia. Nat. Med..

[43] Kurihara M., Matsubara T., Morimoto S., Arakawa A., Ohse K., Kanemaru K., Iwata A., Murayama S., Saito Y. (2024). Neuropathological changes associated with aberrant cerebrospinal fluid p-tau181 and aβ42 in alzheimer’s disease and other neurodegenerative diseases. Acta Neuropathol. Commun..

[44] Graff-Radford J., Yong K.X., Apostolova L.G., Bouwman F.H., Carrillo M., Dickerson B.C., Rabinovici G.D., Schott J.M., Jones D.T., Murray M.E. (2021). New insights into atypical alzheimer’s disease in the era of biomarkers. Lancet Neurol..

[45] Barthélemy N.R., Li Y., Joseph-Mathurin N., Gordon B.A., Hassenstab J., Benzinger T., Buckles V., Fagan A.M., Perrin R.J., Goate A.M. (2020). A soluble phosphorylated tau signature links tau, amyloid and the evolution of stages of dominantly inherited alzheimer’s disease. Nat. Med..

[46] Phan L.M.T., Cho S. (2022). Fluorescent aptasensor and colorimetric aptablot for p-tau231 detection: Toward early diagnosis of alzheimer’s disease. Biomedicines.

[47] Gonzalez-Ortiz F., Turton M., Kac P.R., Smirnov D., Premi E., Ghidoni R., Benussi L., Cantoni V., Saraceno C., Rivolta J. (2023). Brain-derived tau: A novel blood-based biomarker for alzheimer’s disease-type neurodegeneration. Brain.

[48] Cardoso S., Carvalho C., Correia S.C. (2024). Alzheimer’s disease—115 years after its discovery. Biomedicines.

[49] Oeckl P., Halbgebauer S., Anderl-Straub S., von Arnim C.A., Diehl-Schmid J., Froelich L., Grimmer T., Hausner L., Denk J., Jahn H. (2020). Targeted mass spectrometry suggests beta-synuclein as synaptic blood marker in alzheimer’s disease. J. Proteome Res..

[50] Cicognola C., Janelidze S., Hertze J., Zetterberg H., Blennow K., Mattsson-Carlgren N., Hansson O. (2021). Plasma glial fibrillary acidic protein detects alzheimer pathology and predicts future conversion to alzheimer dementia in patients with mild cognitive impairment. Alzheimer’s Res. Ther..

[51] Kaneko N., Nakamura A., Washimi Y., Kato T., Sakurai T., Arahata Y., Bundo M., Takeda A., Niida S., Ito K. (2014). Novel plasma biomarker surrogating cerebral amyloid deposition. Proc. Jpn. Acad. Ser. B.

[52] Pérez-Grijalba V., Arbizu J., Romero J., Prieto E., Pesini P., Sarasa L., Guillen F., Monleón I., San-José I., Martínez-Lage P. (2019). Plasma aβ42/40 ratio alone or combined with fdg-pet can accurately predict amyloid-pet positivity: A cross-sectional analysis from the ab255 study. Alzheimer’s Res. Ther..

[53] Palmqvist S., Janelidze S., Stomrud E., Zetterberg H., Karl J., Zink K., Bittner T., Mattsson N., Eichenlaub U., Blennow K. (2019). Performance of fully automated plasma assays as screening tests for alzheimer disease–related β-amyloid status. JAMA Neurol..

[54] Palmqvist S., Stomrud E., Cullen N., Janelidze S., Manuilova E., Jethwa A., Bittner T., Eichenlaub U., Suridjan I., Kollmorgen G. (2023). An accurate fully automated panel of plasma biomarkers for alzheimer’s disease. Alzheimer’s Dement..

[55] Tropea M.R., Puma D.D.L., Melone M., Gulisano W., Arancio O., Grassi C., Conti F., Puzzo D. (2021). Genetic deletion of α7 nicotinic acetylcholine receptors induces an age-dependent alzheimer’s disease-like pathology. Prog. Neurobiol..

[56] Perluigi M., Di Domenico F., Butterfield D.A. (2024). Oxidative damage in neurodegeneration: Roles in the pathogenesis and progression of alzheimer disease. Physiol. Rev..

[57] Paul P., Bhattacharjee A., Bordoloi S.K., Paul U.K. (2024). The evolution of alzheimer’s disease therapies: A comprehensive review. Ann. Med. Sci. Res..

[58] Fu W.-Y., Ip N.Y. (2023). The role of genetic risk factors of alzheimer’s disease in synaptic dysfunction. Semin. Cell Dev. Biol..

[59] Morabito S., Miyoshi E., Michael N., Swarup V. (2020). Integrative genomics approach identifies conserved transcriptomic networks in alzheimer’s disease. Hum. Mol. Genet..

[60] Kunkle B.W., Grenier-Boley B., Sims R., Bis J.C., Damotte V., Naj A.C., Boland A., Vronskaya M., Van Der Lee S.J., Amlie-Wolf A. (2019). Genetic meta-analysis of diagnosed alzheimer’s disease identifies new risk loci and implicates aβ, tau, immunity and lipid processing. Nat. Genet..

[61] Schwartzentruber J., Cooper S., Liu J.Z., Barrio-Hernandez I., Bello E., Kumasaka N., Young A.M., Franklin R.J., Johnson T., Estrada K. (2021). Genome-wide meta-analysis, fine-mapping and integrative prioritization implicate new alzheimer’s disease risk genes. Nat. Genet..

[62] Escott-Price V., Hardy J. (2022). Genome-wide association studies for alzheimer’s disease: Bigger is not always better. Brain Commun..

[63] Wightman D.P., Jansen I.E., Savage J.E., Shadrin A.A., Bahrami S., Holland D., Rongve A., Børte S., Winsvold B.S., Drange O.K. (2021). A genome-wide association study with 1,126,563 individuals identifies new risk loci for alzheimer’s disease. Nat. Genet..

[64] Deming Y., Li Z., Kapoor M., Harari O., Del-Aguila J.L., Black K., Carrell D., Cai Y., Fernandez M.V., Budde J. (2017). Genome-wide association study identifies four novel loci associated with alzheimer’s endophenotypes and disease modifiers. Acta Neuropathol..

[65] Lee B., Yao X., Shen L. (2022). Genome-wide association study of quantitative biomarkers identifies a novel locus for alzheimer’s disease at 12p12. 1. BMC Genom..

[66] Lambert J.-C., Ibrahim-Verbaas C.A., Harold D., Naj A.C., Sims R., Bellenguez C., DeStafano A., Bis J., Beecham G., Grenier-Boley B. (2013). European alzheimer’s disease initiative (eadi); genetic and environmental risk in alzheimer’s disease; alzheimer’s disease genetic consortium; cohorts for heart and aging research in genomic epidemiology. Meta-analysis of 74,046 individuals identifies 11 new susceptibility loci for alzheimer’s disease. Nat Genet.

[67] Marioni R.E., Harris S.E., Zhang Q., McRae A.F., Hagenaars S.P., Hill W.D., Davies G., Ritchie C.W., Gale C.R., Starr J.M. (2018). Gwas on family history of alzheimer’s disease. Transl. Psychiatry.

[68] Karch C.M., Ezerskiy L.A., Bertelsen S., Consortium A.s.D.G., Goate A.M. (2016). Alzheimer’s disease risk polymorphisms regulate gene expression in the zcwpw1 and the celf1 loci. PLoS ONE.

[69] Karch C.M., Goate A.M. (2015). Alzheimer’s disease risk genes and mechanisms of disease pathogenesis. Biol. Psychiatry.

[70] Kong F., Wu T., Dai J., Cai J., Zhai Z., Zhu Z., Xu Y., Sun T. (2024). Knowledge domains and emerging trends of genome-wide association studies in alzheimer’s disease: A bibliometric analysis and visualization study from 2002 to 2022. PLoS ONE.

[71] Zhu B., Wang R.M., Wang J.T., Chen R.L., Zheng Y.F., Zhang L., Zhao Z.G. (2017). Correlation of rs9331888 polymorphism with alzheimer’s disease among caucasian and chinese populations: A meta-analysis and systematic review. Metab. Brain Dis..

[72] Bellenguez C., Küçükali F., Jansen I.E., Kleineidam L., Moreno-Grau S., Amin N., Naj A.C., Campos-Martin R., Grenier-Boley B., Andrade V. (2022). New insights into the genetic etiology of alzheimer’s disease and related dementias. Nat. Genet..

[73] Iwai K. (2021). Lubac-mediated linear ubiquitination: A crucial regulator of immune signaling. Proc. Jpn. Acad. Ser. B.

[74] Pathak G.A., Silzer T.K., Sun J., Zhou Z., Daniel A.A., Johnson L., O’Bryant S., Phillips N.R., Barber R.C. (2019). Genome-wide methylation of mild cognitive impairment in mexican americans highlights genes involved in synaptic transport, alzheimer’s disease-precursor phenotypes, and metabolic morbidities. J. Alzheimer’s Dis..

[75] Yong W.-S., Hsu F.-M., Chen P.-Y. (2016). Profiling genome-wide DNA methylation. Epigenetics Chromatin.

[76] Sabarwal A., Kumar K., Singh R.P. (2018). Hazardous effects of chemical pesticides on human health–cancer and other associated disorders. Environ. Toxicol. Pharmacol..

[77] Gao X., Chen Q., Yao H., Tan J., Liu Z., Zhou Y., Zou Z. (2022). Epigenetics in alzheimer’s disease. Front. Aging Neurosci..

[78] Andrews S.J., Renton A.E., Fulton-Howard B., Podlesny-Drabiniok A., Marcora E., Goate A.M. (2023). The complex genetic architecture of alzheimer’s disease: Novel insights and future directions. EBioMedicine.

[79] Lee T., Lee H., Initiative A.S.D.N. (2021). Identification of disease-related genes that are common between alzheimer’s and cardiovascular disease using blood genome-wide transcriptome analysis. Biomedicines.

[80] Xu M., Zhang D.-F., Luo R., Wu Y., Zhou H., Kong L.-L., Bi R., Yao Y.-G. (2018). A systematic integrated analysis of brain expression profiles reveals yap1 and other prioritized hub genes as important upstream regulators in alzheimer’s disease. Alzheimer’s Dement..

[81] Dumanis S.B., DiBattista A.M., Miessau M., Moussa C.E., Rebeck G.W. (2013). Apoe genotype affects the pre-synaptic compartment of glutamatergic nerve terminals. J. Neurochem..

[82] Li X., Zhang J., Li D., He C., He K., Xue T., Wan L., Zhang C., Liu Q. (2021). Astrocytic apoe reprograms neuronal cholesterol metabolism and histone-acetylation-mediated memory. Neuron.

[83] Lin Y.-T., Seo J., Gao F., Feldman H.M., Wen H.-L., Penney J., Cam H.P., Gjoneska E., Raja W.K., Cheng J. (2018). Apoe4 causes widespread molecular and cellular alterations associated with alzheimer’s disease phenotypes in human ipsc-derived brain cell types. Neuron.

[84] Litvinchuk A., Huynh T.P.V., Shi Y., Jackson R.J., Finn M.B., Manis M., Francis C.M., Tran A.C., Sullivan P.M., Ulrich J.D. (2021). Apolipoprotein e4 reduction with antisense oligonucleotides decreases neurodegeneration in a tauopathy model. Ann. Neurol..

[85] Marcello E., Saraceno C., Musardo S., Vara H., de La Fuente A.G., Pelucchi S., Di Marino D., Borroni B., Tramontano A., Pérez-Otaño I. (2013). Endocytosis of synaptic adam10 in neuronal plasticity and alzheimer’s disease. J. Clin. Investig..

[86] Kuhn P.-H., Colombo A.V., Schusser B., Dreymueller D., Wetzel S., Schepers U., Herber J., Ludwig A., Kremmer E., Montag D. (2016). Systematic substrate identification indicates a central role for the metalloprotease adam10 in axon targeting and synapse function. Elife.

[87] De Roeck A., Van Broeckhoven C., Sleegers K. (2019). The role of abca7 in alzheimer’s disease: Evidence from genomics, transcriptomics and methylomics. Acta Neuropathol..

[88] Dib S., Pahnke J., Gosselet F. (2021). Role of abca7 in human health and in alzheimer’s disease. Int. J. Mol. Sci..

[89] Yi B., Jahangir A., Evans A.K., Briggs D., Ravina K., Ernest J., Farimani A.B., Sun W., Rajadas J., Green M. (2017). Discovery of novel brain permeable and g protein-biased beta-1 adrenergic receptor partial agonists for the treatment of neurocognitive disorders. PLoS ONE.

[90] Ubelmann F., Burrinha T., Salavessa L., Gomes R., Ferreira C., Moreno N., Guimas Almeida C. (2017). Bin1 and cd 2 ap polarise the endocytic generation of beta-amyloid. EMBO Rep..

[91] Calafate S., Flavin W., Verstreken P., Moechars D. (2016). Loss of bin1 promotes the propagation of tau pathology. Cell Rep..

[92] Crotti A., Sait H.R., McAvoy K.M., Estrada K., Ergun A., Szak S., Marsh G., Jandreski L., Peterson M., Reynolds T.L. (2019). Bin1 favors the spreading of tau via extracellular vesicles. Sci. Rep..

[93] Yu Y., Niccoli T., Ren Z., Woodling N.S., Aleyakpo B., Szabadkai G., Partridge L. (2020). Picalm rescues glutamatergic neurotransmission, behavioural function and survival in a drosophila model of aβ42 toxicity. Hum. Mol. Genet..

[94] Crehan H., Holton P., Wray S., Pocock J., Guerreiro R., Hardy J. (2012). Complement receptor 1 (cr1) and alzheimer’s disease. Immunobiology.

[95] Adams C.J., Kopp M.C., Larburu N., Nowak P.R., Ali M.M. (2019). Structure and molecular mechanism of er stress signaling by the unfolded protein response signal activator ire1. Front. Mol. Biosci..

[96] Duran-Aniotz C., Cornejo V.H., Espinoza S., Ardiles Á.O., Medinas D.B., Salazar C., Foley A., Gajardo I., Thielen P., Iwawaki T. (2017). Ire1 signaling exacerbates alzheimer’s disease pathogenesis. Acta Neuropathol..

[97] Wang Y., Fu W.-Y., Cheung K., Hung K.-W., Chen C., Geng H., Yung W.-H., Qu J.Y., Fu A.K., Ip N.Y. (2021). Astrocyte-secreted il-33 mediates homeostatic synaptic plasticity in the adult hippocampus. Proc. Natl. Acad. Sci. USA.

[98] Djurisic M., Brott B.K., Saw N.L., Shamloo M., Shatz C.J. (2019). Activity-dependent modulation of hippocampal synaptic plasticity via pirb and endocannabinoids. Mol. Psychiatry.

[99] Barić N. (2022). The decline of the expression of low density lipoprotein receptor-related protein 1 (lrp1) during normal ageing and in alzheimer’s disease. Glycative Stress Res..

[100] Belinson H., Lev D., Masliah E., Michaelson D.M. (2008). Activation of the amyloid cascade in apolipoprotein e4 transgenic mice induces lysosomal activation and neurodegeneration resulting in marked cognitive deficits. J. Neurosci..

[101] Candela P., Saint-Pol J., Kuntz M., Boucau M.-C., Lamartiniere Y., Gosselet F., Fenart L. (2015). In vitro discrimination of the role of lrp1 at the bbb cellular level: Focus on brain capillary endothelial cells and brain pericytes. Brain Res..

[102] Deane R., Wu Z., Sagare A., Davis J., Du Yan S., Hamm K., Xu F., Parisi M., LaRue B., Hu H.W. (2004). Lrp/amyloid β-peptide interaction mediates differential brain efflux of aβ isoforms. Neuron.

[103] Shinohara M., Tachibana M., Kanekiyo T., Bu G. (2017). Role of lrp1 in the pathogenesis of alzheimer’s disease: Evidence from clinical and preclinical studies: Thematic review series: Apoe and lipid homeostasis in alzheimer’s disease. J. Lipid Res..

[104] Hsin H., Kim M.J., Wang C.-F., Sheng M. (2010). Proline-rich tyrosine kinase 2 regulates hippocampal long-term depression. J. Neurosci..

[105] de Pins B., Mendes T., Giralt A., Girault J.-A. (2021). The non-receptor tyrosine kinase pyk2 in brain function and neurological and psychiatric diseases. Front. Synaptic Neurosci..

[106] Nackenoff A.G., Hohman T.J., Neuner S.M., Akers C.S., Weitzel N.C., Shostak A., Ferguson S.M., Mobley B., Bennett D.A., Schneider J.A. (2021). Pld3 is a neuronal lysosomal phospholipase d associated with β-amyloid plaques and cognitive function in alzheimer’s disease. PLoS Genet..

[107] Radford H., Moreno J.A., Verity N., Halliday M., Mallucci G.R. (2015). Perk inhibition prevents tau-mediated neurodegeneration in a mouse model of frontotemporal dementia. Acta Neuropathol..

[108] Yuan S.H., Hiramatsu N., Liu Q., Sun X.V., Lenh D., Chan P., Chiang K., Koo E.H., Kao A.W., Litvan I. (2018). Tauopathy-associated perk alleles are functional hypomorphs that increase neuronal vulnerability to er stress. Hum. Mol. Genet..

[109] Schmidt V., Subkhangulova A., Willnow T.E. (2017). Sorting receptor sorla: Cellular mechanisms and implications for disease. Cell. Mol. Life Sci..

[110] Malik A.R., Willnow T.E. (2020). Vps10p domain receptors: Sorting out brain health and disease. Trends Neurosci..

[111] Ulland T.K., Colonna M. (2018). Trem2—A key player in microglial biology and alzheimer disease. Nat. Rev. Neurol..

[112] Deczkowska A., Weiner A., Amit I. (2020). The physiology, pathology, and potential therapeutic applications of the trem2 signaling pathway. Cell.

[113] Di Paolo G., Kim T.-W. (2011). Linking lipids to alzheimer’s disease: Cholesterol and beyond. Nat. Rev. Neurosci..

[114] González A., Calfío C., Churruca M., Maccioni R.B. (2022). Glucose metabolism and ad: Evidence for a potential diabetes type 3. Alzheimer’s Res. Ther..

[115] Akhtar A., Sah S.P. (2020). Insulin signaling pathway and related molecules: Role in neurodegeneration and alzheimer’s disease. Neurochem. Int..

[116] Perluigi M., Di Domenico F., Butterfield D.A. (2015). Mtor signaling in aging and neurodegeneration: At the crossroad between metabolism dysfunction and impairment of autophagy. Neurobiol. Dis..

[117] Urbanska M., Gozdz A., Swiech L.J., Jaworski J. (2012). Mammalian target of rapamycin complex 1 (mtorc1) and 2 (mtorc2) control the dendritic arbor morphology of hippocampal neurons. J. Biol. Chem..

[118] Banks W.A., Owen J.B., Erickson M.A. (2012). Insulin in the brain: There and back again. Pharmacol. Ther..

[119] Neth B.J., Craft S. (2017). Insulin resistance and alzheimer’s disease: Bioenergetic linkages. Front. Aging Neurosci..

[120] Zhou S., Tu L., Chen W., Yan G., Guo H., Wang X., Hu Q., Liu H., Li F. (2024). Alzheimer’s disease, a metabolic disorder: Clinical advances and basic model studies. Exp. Ther. Med..

[121] Stanciu G.D., Rusu R.N., Bild V., Filipiuc L.E., Tamba B.-I., Ababei D.C. (2021). Systemic actions of sglt2 inhibition on chronic mtor activation as a shared pathogenic mechanism between alzheimer’s disease and diabetes. Biomedicines.

[122] Rosa E., Fahnestock M. (2015). Creb expression mediates amyloid β-induced basal bdnf downregulation. Neurobiol. Aging.

[123] Cheng Y., Tian D.-Y., Wang Y.-J. (2020). Peripheral clearance of brain-derived aβ in alzheimer’s disease: Pathophysiology and therapeutic perspectives. Transl. Neurodegener..

[124] Fahnestock M. (2011). Brain-derived neurotrophic factor: The link between amyloid-β and memory loss. Future Neurol..

[125] Campos-Pena V., Toral-Rios D., Becerril-Pérez F., Sánchez-Torres C., Delgado-Namorado Y., Torres-Ossorio E., Franco-Bocanegra D., Carvajal K. (2017). Metabolic syndrome as a risk factor for alzheimer’s disease: Is aβ a crucial factor in both pathologies?. Antioxid. Redox Signal..

[126] Chen Z., Zhong C. (2014). Oxidative stress in alzheimer’s disease. Neurosci. Bull..

[127] Ioannou M.S., Jackson J., Sheu S.-H., Chang C.-L., Weigel A.V., Liu H., Pasolli H.A., Xu C.S., Pang S., Matthies D. (2019). Neuron-astrocyte metabolic coupling protects against activity-induced fatty acid toxicity. Cell.

[128] Fazzari P., Horre K., Arranz A.M., Frigerio C.S., Saito T., Saido T.C., De Strooper B. (2017). Pld3 gene and processing of app. Nature.

[129] Cai R., Wang Y., Huang Z., Zou Q., Pu Y., Yu C., Cai Z. (2021). Role of rhoa/rock signaling in alzheimer’s disease. Behav. Brain Res..

[130] Xiang X., Wang X., Jin S., Hu J., Wu Y., Li Y., Wu X. (2022). Activation of gpr55 attenuates cognitive impairment and neurotoxicity in a mouse model of alzheimer’s disease induced by aβ1–42 through inhibiting rhoa/rock2 pathway. Prog. Neuro-Psychopharmacol. Biol. Psychiatry.

[131] Van Eldik L.J., Carrillo M.C., Cole P.E., Feuerbach D., Greenberg B.D., Hendrix J.A., Kennedy M., Kozauer N., Margolin R.A., Molinuevo J.L. (2016). The roles of inflammation and immune mechanisms in alzheimer’s disease. Alzheimer’s Dement. Transl. Res. Clin. Interv..

[132] Shi J.-Q., Shen W., Chen J., Wang B.-R., Zhong L.-L., Zhu Y.-W., Zhu H.-Q., Zhang Q.-Q., Zhang Y.-D., Xu J. (2011). Anti-tnf-α reduces amyloid plaques and tau phosphorylation and induces cd11c-positive dendritic-like cell in the app/ps1 transgenic mouse brains. Brain Res..

[133] Nguyen H.D., Vu H.H. (2024). Molecular mechanisms implicated in protein changes in the alzheimer’s disease human hippocampus. Mech. Ageing Dev..

[134] Wang Y., Li L., Wu Y., Zhang S., Ju Q., Yang Y., Jin Y., Shi H., Sun C. (2022). Cd44 deficiency represses neuroinflammation and rescues dopaminergic neurons in a mouse model of parkinson’s disease. Pharmacol. Res..

[135] Smith R., Klein P., Koc-Schmitz Y., Waldvogel H.J., Faull R.L., Brundin P., Plomann M., Li J.Y. (2007). Loss of snap-25 and rabphilin 3a in sensory-motor cortex in huntington’s disease. J. Neurochem..

[136] Wu M., Fang K., Wang W., Lin W., Guo L., Wang J. (2019). Identification of key genes and pathways for alzheimer’s disease via combined analysis of genome-wide expression profiling in the hippocampus. Biophys. Rep..

[137] Ali M., Falkenhain K., Njiru B.N., Murtaza-Ali M., Ruiz-Uribe N.E., Haft-Javaherian M., Catchers S., Nishimura N., Schaffer C.B., Bracko O. (2022). Vegf signalling causes stalls in brain capillaries and reduces cerebral blood flow in alzheimer’s mice. Brain.

[138] García-Culebras A., Cuartero M.I., Peña-Martínez C., Moraga A., Vázquez-Reyes S., de Castro-Millán F.J., Cortes-Canteli M., Lizasoain I., Moro M.Á. (2024). Myeloid cells in vascular dementia and alzheimer’s disease: Possible therapeutic targets?. Br. J. Pharmacol..

[139] Kang L., Yu H., Yang X., Zhu Y., Bai X., Wang R., Cao Y., Xu H., Luo H., Lu L. (2020). Neutrophil extracellular traps released by neutrophils impair revascularization and vascular remodeling after stroke. Nat. Commun..

[140] Cai Z., Wan C.-Q., Liu Z. (2017). Astrocyte and alzheimer’s disease. J. Neurol..

[141] Fernandez C.G., Hamby M.E., McReynolds M.L., Ray W.J. (2019). The role of apoe4 in disrupting the homeostatic functions of astrocytes and microglia in aging and alzheimer’s disease. Front. Aging Neurosci..

[142] Hickman S.E., El Khoury J. (2014). Trem2 and the neuroimmunology of alzheimer’s disease. Biochem. Pharmacol..

[143] Kleinberger G., Yamanishi Y., Suárez-Calvet M., Czirr E., Lohmann E., Cuyvers E., Struyfs H., Pettkus N., Wenninger-Weinzierl A., Mazaheri F. (2014). Trem2 mutations implicated in neurodegeneration impair cell surface transport and phagocytosis. Sci. Transl. Med..

[144] Guerreiro R., Wojtas A., Bras J., Carrasquillo M., Rogaeva E., Majounie E., Cruchaga C., Sassi C., Kauwe J.S., Younkin S. (2013). Trem2 variants in alzheimer’s disease. N. Engl. J. Med..

[145] Steinberg S., Stefansson H., Jonsson T., Johannsdottir H., Ingason A., Helgason H., Sulem P., Magnusson O.T., Gudjonsson S.A., Unnsteinsdottir U. (2015). Loss-of-function variants in abca7 confer risk of alzheimer’s disease. Nat. Genet..

[146] Lian H., Yang L., Cole A., Sun L., Chiang A.C.-A., Fowler S.W., Shim D.J., Rodriguez-Rivera J., Taglialatela G., Jankowsky J.L. (2015). Nfκb-activated astroglial release of complement c3 compromises neuronal morphology and function associated with alzheimer’s disease. Neuron.

[147] Jayaraman A., Htike T.T., James R., Picon C., Reynolds R. (2021). Tnf-mediated neuroinflammation is linked to neuronal necroptosis in alzheimer’s disease hippocampus. Acta Neuropathol. Commun..

[148] Picon C., Jayaraman A., James R., Beck C., Gallego P., Witte M.E., van Horssen J., Mazarakis N.D., Reynolds R. (2021). Neuron-specific activation of necroptosis signaling in multiple sclerosis cortical grey matter. Acta Neuropathol..

[149] Asimakidou E., Reynolds R., Barron A.M., Lo C.H. (2024). Autolysosomal acidification impairment as a mediator for tnfr1 induced neuronal necroptosis in alzheimer’s disease. Neural Regen. Res..

[150] Song L., Pei L., Yao S., Wu Y., Shang Y. (2017). Nlrp3 inflammasome in neurological diseases, from functions to therapies. Front. Cell. Neurosci..

[151] Tripathi P., Rodriguez-Muela N., Klim J.R., de Boer A.S., Agrawal S., Sandoe J., Lopes C.S., Ogliari K.S., Williams L.A., Shear M. (2017). Reactive astrocytes promote als-like degeneration and intracellular protein aggregation in human motor neurons by disrupting autophagy through tgf-β1. Stem Cell Rep..

[152] Feng T., Lacrampe A., Hu F. (2021). Physiological and pathological functions of tmem106b: A gene associated with brain aging and multiple brain disorders. Acta Neuropathol..

[153] Chandrasekaran A., Dittlau K.S., Corsi G.I., Haukedal H., Doncheva N.T., Ramakrishna S., Ambardar S., Salcedo C., Schmidt S.I., Zhang Y. (2021). Astrocytic reactivity triggered by defective autophagy and metabolic failure causes neurotoxicity in frontotemporal dementia type 3. Stem Cell Rep..

[154] Iyer H., Shen K., Meireles A.M., Talbot W.S. (2022). A lysosomal regulatory circuit essential for the development and function of microglia. Sci. Adv..

[155] Jayaraman A., Reynolds R. (2022). Diverse pathways to neuronal necroptosis in alzheimer’s disease. Eur. J. Neurosci..

[156] Udeochu J.C., Shea J.M., Villeda S.A. (2016). Microglia communication: Parallels between aging and alzheimer’s disease. Clin. Exp. Neuroimmunol..

[157] Lee J., Chan S.L., Mattson M.P. (2002). Adverse effect of a presenilin-1 mutation in microglia results in enhanced nitric oxide and inflammatory cytokine responses to immune challenge in the brain. Neuromol. Med..

[158] Fung S., Smith C.L., Prater K.E., Case A., Green K., Osnis L., Winston C., Kinoshita Y., Sopher B., Morrison R.S. (2020). Early-onset familial alzheimer disease variant psen2 n141i heterozygosity is associated with altered microglia phenotype. J. Alzheimer’s Dis..

[159] Deus C.M., Yambire K.F., Oliveira P.J., Raimundo N. (2020). Mitochondria–lysosome crosstalk: From physiology to neurodegeneration. Trends Mol. Med..

[160] Quick J.D., Silva C., Wong J.H., Lim K.L., Reynolds R., Barron A.M., Zeng J., Lo C.H. (2023). Lysosomal acidification dysfunction in microglia: An emerging pathogenic mechanism of neuroinflammation and neurodegeneration. J. Neuroinflamm..

[161] Vest R.T., Chou C.-C., Zhang H., Haney M.S., Li L., Laqtom N.N., Chang B., Shuken S., Nguyen A., Yerra L. (2022). Small molecule c381 targets the lysosome to reduce inflammation and ameliorate disease in models of neurodegeneration. Proc. Natl. Acad. Sci. USA.

[162] Chen C.-C., Keller M., Hess M., Schiffmann R., Urban N., Wolfgardt A., Schaefer M., Bracher F., Biel M., Wahl-Schott C. (2014). A small molecule restores function to trpml1 mutant isoforms responsible for mucolipidosis type iv. Nat. Commun..

[163] Tong B.C.-K., Huang A.S., Wu A.J., Iyaswamy A., Ho O.K.-Y., Kong A.H.-Y., Sreenivasmurthy S.G., Zhu Z., Su C., Liu J. (2022). Tetrandrine ameliorates cognitive deficits and mitigates tau aggregation in cell and animal models of tauopathies. J. Biomed. Sci..

[164] Yao X.C., Xue X., Zhang H.T., Zhu M.M., Yang X.W., Wu C.F., Yang J.Y. (2019). Pseudoginsenoside-f11 alleviates oligomeric β-amyloid-induced endosome-lysosome defects in microglia. Traffic.

[165] Chin M.Y., Ang K.-H., Davies J., Alquezar C., Garda V.G., Rooney B., Leng K., Kampmann M., Arkin M.R., Kao A.W. (2022). Phenotypic screening using high-content imaging to identify lysosomal ph modulators in a neuronal cell model. ACS Chem. Neurosci..

[166] Zeng J., Shirihai O.S., Grinstaff M.W. (2020). Modulating lysosomal ph: A molecular and nanoscale materials design perspective. J. Life Sci..

[167] Brouillard M., Barthélémy P., Dehay B., Crauste-Manciet S., Desvergnes V. (2021). Nucleolipid acid-based nanocarriers restore neuronal lysosomal acidification defects. Front. Chem..

[168] Cunha A., Gaubert A., Latxague L., Dehay B. (2021). Plga-based nanoparticles for neuroprotective drug delivery in neurodegenerative diseases. Pharmaceutics.

[169] Lo C.H., Zeng J. (2023). Defective lysosomal acidification: A new prognostic marker and therapeutic target for neurodegenerative diseases. Transl. Neurodegener..

[170] Frost G.R., Li Y.-M. (2017). The role of astrocytes in amyloid production and alzheimer’s disease. Open Biol..

[171] Jablonski A.M., Warren L., Usenovic M., Zhou H., Sugam J., Parmentier-Batteur S., Voleti B. (2021). Astrocytic expression of the alzheimer’s disease risk allele, apoeε4, potentiates neuronal tau pathology in multiple preclinical models. Sci. Rep..

[172] Rojas-Gutierrez E., Muñoz-Arenas G., Treviño S., Espinosa B., Chavez R., Rojas K., Flores G., Díaz A., Guevara J. (2017). Alzheimer’s disease and metabolic syndrome: A link from oxidative stress and inflammation to neurodegeneration. Synapse.

[173] Ni J., Xie Z., Quan Z., Meng J., Qing H. (2024). How brain ‘cleaners’ fail: Mechanisms and therapeutic value of microglial phagocytosis in alzheimer’s disease. Glia.

[174] Mancuso R., Fryatt G., Cleal M., Obst J., Pipi E., Monzón-Sandoval J., Ribe E., Winchester L., Webber C., Nevado A. (2019). Csf1r inhibitor jnj-40346527 attenuates microglial proliferation and neurodegeneration in p301s mice. Brain.

[175] Chen J., Zhou Y., Mueller-Steiner S., Chen L.-F., Kwon H., Yi S., Mucke L., Gan L. (2005). Sirt1 protects against microglia-dependent amyloid-β toxicity through inhibiting nf-κb signaling. J. Biol. Chem..

[176] Wang C., Fan L., Khawaja R.R., Liu B., Zhan L., Kodama L., Chin M., Li Y., Le D., Zhou Y. (2022). Microglial nf-κb drives tau spreading and toxicity in a mouse model of tauopathy. Nat. Commun..

[177] Yan S.D., Bierhaus A., Nawroth P.P., Stern D.M. (2009). Rage and alzheimer’s disease: A progression factor for amyloid-β-induced cellular perturbation?. J. Alzheimer’s Dis..

[178] Farmer D.G., Ewart M.-A., Mair K.M., Kennedy S. (2014). Soluble receptor for advanced glycation end products (srage) attenuates haemodynamic changes to chronic hypoxia in the mouse. Pulm. Pharmacol. Ther..

[179] Heilmann R.M., Otoni C.C., Jergens A.E., Grützner N., Suchodolski J.S., Steiner J.M. (2014). Systemic levels of the anti-inflammatory decoy receptor soluble rage (receptor for advanced glycation end products) are decreased in dogs with inflammatory bowel disease. Vet. Immunol. Immunopathol..

[180] Kook S.-Y., Seok Hong H., Moon M., Mook-Jung I. (2013). Disruption of blood-brain barrier in alzheimer disease pathogenesis. Tissue Barriers.

[181] Wan W., Chen H., Li Y. (2014). The potential mechanisms of aβ-receptor for advanced glycation end-products interaction disrupting tight junctions of the blood-brain barrier in alzheimer’s disease. Int. J. Neurosci..

[182] Patel M. (2024). Implications of the clearance methods for amyloid-beta plaques in alzheimer’s disease. Int. J. High Sch. Res..

[183] Munoz L., Ranaivo H.R., Roy S.M., Hu W., Craft J.M., McNamara L.K., Chico L.W., Van Eldik L.J., Watterson D.M. (2007). A novel p38α mapk inhibitor suppresses brain proinflammatory cytokine up-regulation and attenuates synaptic dysfunction and behavioral deficits in an alzheimer’s disease mouse model. J. Neuroinflamm..

[184] Puzzo D., Privitera L., Leznik E., Fa M., Staniszewski A., Palmeri A., Arancio O. (2008). Picomolar amyloid-β positively modulates synaptic plasticity and memory in hippocampus. J. Neurosci..

[185] Arancio O., Zhang H.P., Chen X., Lin C., Trinchese F., Puzzo D., Liu S., Hegde A., Yan S.F., Stern A. (2004). Rage potentiates aβ-induced perturbation of neuronal function in transgenic mice. EMBO J..

[186] Vincenz-Donnelly L., Hipp M.S. (2017). The endoplasmic reticulum: A hub of protein quality control in health and disease. Free Radic. Biol. Med..

[187] Fregno I., Molinari M. (2019). Proteasomal and lysosomal clearance of faulty secretory proteins: Er-associated degradation (erad) and er-to-lysosome-associated degradation (erlad) pathways. Crit. Rev. Biochem. Mol. Biol..

[188] Xu S., Liu H., Wang C., Deng Y., Xu B., Yang T., Liu W. (2023). Dual roles of uprer and uprmt in neurodegenerative diseases. J. Mol. Med..

[189] Montibeller L., De Belleroche J. (2018). Amyotrophic lateral sclerosis (als) and alzheimer’s disease (ad) are characterised by differential activation of er stress pathways: Focus on upr target genes. Cell Stress Chaperones.

[190] Yu Z., Dou F., Wang Y., Hou L., Chen H. (2018). Ca^2+^-dependent endoplasmic reticulum stress correlation with astrogliosis involves upregulation of kca3. 1 and inhibition of akt/mtor signaling. J. Neuroinflamm..

[191] Wan Y.-W., Al-Ouran R., Mangleburg C.G., Perumal T.M., Lee T.V., Allison K., Swarup V., Funk C.C., Gaiteri C., Allen M. (2020). Meta-analysis of the alzheimer’s disease human brain transcriptome and functional dissection in mouse models. Cell Rep..

[192] Cissé M., Duplan E., Lorivel T., Dunys J., Bauer C., Meckler X., Gerakis Y., Lauritzen I., Checler F. (2017). The transcription factor xbp1s restores hippocampal synaptic plasticity and memory by control of the kalirin-7 pathway in alzheimer model. Mol. Psychiatry.

[193] Cozachenco D., Ribeiro F.C., Ferreira S.T. (2023). Defective proteostasis in alzheimer’s disease. Ageing Res. Rev..

[194] Ahn J.H., Cho H., Kim J.-H., Kim S.H., Ham J.-S., Park I., Suh S.H., Hong S.P., Song J.-H., Hong Y.-K. (2019). Meningeal lymphatic vessels at the skull base drain cerebrospinal fluid. Nature.

[195] Storck S.E., Meister S., Nahrath J., Meißner J.N., Schubert N., Di Spiezio A., Baches S., Vandenbroucke R.E., Bouter Y., Prikulis I. (2016). Endothelial lrp1 transports amyloid-β 1–42 across the blood-brain barrier. J. Clin. Investig..

[196] Soto I., Grabowska W.A., Onos K.D., Graham L.C., Jackson H.M., Simeone S.N., Howell G.R. (2016). Meox2 haploinsufficiency increases neuronal cell loss in a mouse model of alzheimer’s disease. Neurobiol. Aging.

[197] Nixon R.A., Yang D.-S. (2011). Autophagy failure in alzheimer’s disease—Locating the primary defect. Neurobiol. Dis..

[198] Nilsson P., Loganathan K., Sekiguchi M., Matsuba Y., Hui K., Tsubuki S., Tanaka M., Iwata N., Saito T., Saido T.C. (2013). Aβ secretion and plaque formation depend on autophagy. Cell Rep..

[199] Nilsson P., Sekiguchi M., Akagi T., Izumi S., Komori T., Hui K., Sörgjerd K., Tanaka M., Saito T., Iwata N. (2015). Autophagy-related protein 7 deficiency in amyloid β (aβ) precursor protein transgenic mice decreases aβ in the multivesicular bodies and induces aβ accumulation in the golgi. Am. J. Pathol..

[200] Hara T., Nakamura K., Matsui M., Yamamoto A., Nakahara Y., Suzuki-Migishima R., Yokoyama M., Mishima K., Saito I., Okano H. (2006). Suppression of basal autophagy in neural cells causes neurodegenerative disease in mice. Nature.

[201] Komatsu M., Waguri S., Chiba T., Murata S., Iwata J.-i., Tanida I., Ueno T., Koike M., Uchiyama Y., Kominami E. (2006). Loss of autophagy in the central nervous system causes neurodegeneration in mice. Nature.

[202] Harris H., Rubinsztein D.C. (2012). Control of autophagy as a therapy for neurodegenerative disease. Nat. Rev. Neurol..

[203] Liu J., Li L. (2019). Targeting autophagy for the treatment of alzheimer’s disease: Challenges and opportunities. Front. Mol. Neurosci..

[204] Du Y., Wooten M.C., Gearing M., Wooten M.W. (2009). Age-associated oxidative damage to the p62 promoter: Implications for alzheimer disease. Free Radic. Biol. Med..

[205] Rohn T.T., Wirawan E., Brown R.J., Harris J.R., Masliah E., Vandenabeele P. (2011). Depletion of beclin-1 due to proteolytic cleavage by caspases in the alzheimer’s disease brain. Neurobiol. Dis..

[206] Esteves A., Palma A., Gomes R., Santos D., Silva D., Cardoso S. (2019). Acetylation as a major determinant to microtubule-dependent autophagy: Relevance to alzheimer’s and parkinson disease pathology. Biochim. Et Biophys. Acta (BBA) Mol. Basis Dis..

[207] Sola-Sevilla N., Puerta E. (2024). Sirt2 as a potential new therapeutic target for alzheimer’s disease. Neural Regen. Res..

[208] Alamro H., Thafar M.A., Albaradei S., Gojobori T., Essack M., Gao X. (2023). Exploiting machine learning models to identify novel alzheimer’s disease biomarkers and potential targets. Sci. Rep..

[209] Long J.M., Holtzman D.M. (2019). Alzheimer disease: An update on pathobiology and treatment strategies. Cell.

[210] Wojtunik-Kulesza K., Rudkowska M., Orzeł-Sajdłowska A. (2023). Aducanumab—Hope or disappointment for alzheimer’s disease. Int. J. Mol. Sci..

[211] Van Dyck C.H., Swanson C.J., Aisen P., Bateman R.J., Chen C., Gee M., Kanekiyo M., Li D., Reyderman L., Cohen S. (2023). Lecanemab in early alzheimer’s disease. N. Engl. J. Med..

[212] Cummings J., Zhou Y., Lee G., Zhong K., Fonseca J., Cheng F. (2023). Alzheimer’s disease drug development pipeline: 2023. Alzheimer’s Dement. Transl. Res. Clin. Interv..

[213] Jadhav D., Saraswat N., Vyawahare N., Shirode D. (2024). Targeting the molecular web of alzheimer’s disease: Unveiling pathways for effective pharmacotherapy. Egypt. J. Neurol. Psychiatry Neurosurg..

[214] Peng Y., Jin H., Xue Y.-h., Chen Q., Yao S.-y., Du M.-q., Liu S. (2023). Current and future therapeutic strategies for alzheimer’s disease: An overview of drug development bottlenecks. Front. Aging Neurosci..

[215] Jones R.S., Minogue A.M., Fitzpatrick O., Lynch M.A. (2015). Inhibition of jak2 attenuates the increase in inflammatory markers in microglia from app/ps1 mice. Neurobiol. Aging.

[216] Arnold S.E., Arvanitakis Z., Macauley-Rambach S.L., Koenig A.M., Wang H.-Y., Ahima R.S., Craft S., Gandy S., Buettner C., Stoeckel L.E. (2018). Brain insulin resistance in type 2 diabetes and alzheimer disease: Concepts and conundrums. Nat. Rev. Neurol..

[217] Abolhassani N., Leon J., Sheng Z., Oka S., Hamasaki H., Iwaki T., Nakabeppu Y. (2017). Molecular pathophysiology of impaired glucose metabolism, mitochondrial dysfunction, and oxidative DNA damage in alzheimer’s disease brain. Mech. Ageing Dev..

[218] Butterfield D.A., Halliwell B. (2019). Oxidative stress, dysfunctional glucose metabolism and alzheimer disease. Nat. Rev. Neurosci..

[219] Lee J.-H., Yang D.-S., Goulbourne C.N., Im E., Stavrides P., Pensalfini A., Chan H., Bouchet-Marquis C., Bleiwas C., Berg M.J. (2022). Faulty autolysosome acidification in alzheimer’s disease mouse models induces autophagic build-up of aβ in neurons, yielding senile plaques. Nat. Neurosci..

